# A Double-Track Pathway to Fast Strategy in Humans and Its Personality Correlates

**DOI:** 10.3389/fpsyg.2022.889730

**Published:** 2022-06-09

**Authors:** Fernando Gutiérrez, Josep M. Peri, Eva Baillès, Bárbara Sureda, Miguel Gárriz, Gemma Vall, Myriam Cavero, Aida Mallorquí, José Ruiz Rodríguez

**Affiliations:** ^1^Institute of Neuroscience, Hospital Clínic de Barcelona, Barcelona, Spain; ^2^Institut d’Investigacións Biomèdiques August Pi Sunyer (IDIBAPS), Barcelona, Spain; ^3^Department of Experimental and Health Sciences, Pompeu Fabra University, Barcelona, Spain; ^4^Institut de Neuropsiquiatria i Addiccions (INAD), Parc de Salut Mar, Barcelona, Spain; ^5^Department of Psychiatry, Mental Health, and Addiction, GSS–Hospital Santa Maria, Lleida, Spain; ^6^Lleida Institute for Biomedical Research Dr. Pifarré Foundation, Lleida, Spain; ^7^Department of Clinical Psychology and Psychobiology, Personality, Evaluation and Psychological Treatment Section, Institute of Neurosciences, University of Barcelona, Barcelona, Spain

**Keywords:** fast–slow continuum, life history, personality, personality disorders, evolutionary psychology

## Abstract

The fast–slow paradigm of life history (LH) focuses on how individuals grow, mate, and reproduce at different paces. This paradigm can contribute substantially to the field of personality and individual differences provided that it is more strictly based on evolutionary biology than it has been so far. Our study tested the existence of a fast–slow continuum underlying indicators of reproductive effort—offspring output, age at first reproduction, number and stability of sexual partners—in 1,043 outpatients with healthy to severely disordered personalities. Two axes emerged reflecting a double-track pathway to fast strategy, based on restricted and unrestricted sociosexual strategies. When rotated, the fast–slow and sociosexuality axes turned out to be independent. Contrary to expectations, neither somatic effort—investment in status, material resources, social capital, and maintenance/survival—was aligned with reproductive effort, nor a clear tradeoff between current and future reproduction was evident. Finally, we examined the association of LH axes with seven high-order personality pathology traits: negative emotionality, impulsivity, antagonism, persistence-compulsivity, subordination, and psychoticism. Persistent and disinhibited subjects appeared as fast-restricted and fast-unrestricted strategists, respectively, whereas asocial subjects were slow strategists. Associations of LH traits with each other and with personality are far more complex than usually assumed in evolutionary psychology.

## Introduction

Living things compete to reap energy from the environment and convert it into replicates of themselves. Success in this endeavor—fitness—requires fine decisions on where energy allocations should be made (Stearns, 1992; Roff, 2002; Bolund, 2020). *Reproductive effort* is the most obvious target for investment. In sexual species, it entails *mating effort*, that is, finding the better possible mate, gaining sexual access to it, and carrying through fertilization, gestation, and delivery. In altricial species, with underdeveloped and helpless young, it also involves *parenting effort*, i.e., supporting the survival and success of the resulting offspring. To this aim, however, the organisms need to previously invest energy in *somatic effort*. On the one hand, this means *growth*, the acquisition of the size, strength, and skills that enable the organism to outcompete rivals in gaining access to food, shelter, territory, status, and mates, and to protect the progeny. On the other, energy should be also devoted to *maintenance* and *survival*: remaining alive and in good condition as long as possible in order to maximize reproductive opportunities. This includes repairing tissues against the ravages of time, investing in an immune system, and dealing with biotic and abiotic hazards such as climatic events, famine, infectious agents, predators, or conspecifics.

However, organisms have limited time and energy, and not all fitness components can be maximized at once. Instead, individuals, populations, and species must favor some allocations over others to maximize their genetic contribution to the next generation. This gives place to tradeoffs, i.e., the negative correlation between two fitness components (Roff, 2002). For example, advancing reproduction limits growth and jeopardizes resource storing for the future, increasing offspring output detracts from body maintenance and survival, spending heavily on offspring quality (e.g., through parenting) delays additional reproduction, and adopting a promiscuous mating strategy takes resources away from parental care. Dozens of other less-studied tradeoffs have been identified (Stearns, 1992). In addition, tradeoffs are not functionally independent from one another but covary, resulting in complex networks of intertwined “choices” called life history (LH) strategies. LH theory focuses on how fitness is optimized through different pathways, which depend on where and when energy is allocated across the life course between the different components of reproductive and somatic effort (Stearns, 1992; Bolund, 2020).

At the species level, LH traits seem to be underlain by a broad axis, the fast–slow continuum (Promislow and Harvey, 1990; Healy et al., 2019). Species at the fast pole of the continuum, like rodents or sparrows, show quick growth, small adult body size, early reproduction, high reproductive output, rapid senescence, and short lifespan. Slow strategists, like rhinos or pelicans, invest more in growing and maintaining health, mature slowly, attain larger sizes, reproduce late and rarely, and die at old ages. Essentially, a fast strategy is the prioritization of current over future reproduction, and of mating over any other allocation.

This said, some gaps still need to be addressed regarding the fast–slow paradigm. First of all, there are doubts on the very existence of a fast–slow continuum, or at least on whether the tradeoffs between biodemographic indicators of mating, parenting, and somatic effort consistently take this form across sex, taxa, and organismal and environmental conditions (Bielby et al., 2007; Sear, 2020; Stearns and Rodrigues, 2020). For example, it has been argued that the fast–slow continuum losses some distinctness when body size is controlled for, and it does not emerge as unmistakably in fish, reptiles, or insects as it does in mammals and birds (Stearns, 1983; Bauwens and Díaz-Uriarte, 1997; Bielby et al., 2007; Jeschke and Kokko, 2009; Bakewell et al., 2020). Accordingly, it has been suggested that tradeoffs should be studied independently from one another rather than amalgamated in a single construct (Međedović, 2020a, 2021; Sear, 2020). On the flip side, it has been found that controlling for body size does not make the continuum disappear altogether, that much disparity among taxa may be due to differing axes rotations, and that there is ample evidence that combinations of LH traits are relatively constrained and consistently adopt the form of a fast–slow continuum (Stearns, 1983; Bielby et al., 2007; Dobson and Oli, 2007; Salguero-Gómez et al., 2016; Healy et al., 2019; Del Giudice, 2020; Woodley Menie et al., 2021). Whatever the case, there is agreement that a single axis is insufficient to explain the wide range of LH variation. Two axes have been often found, and there might be three or more (Bielby et al., 2007; Cooke et al., 2019; Healy et al., 2019; Stearns and Rodrigues, 2020).

Secondly, there is doubt as to whether the fast–slow continuum, initially proposed to explain interspecies differences, also exists among individuals of the same species. In this regard, it has been argued that interspecies and interindividual differences derive from different mechanisms and their underlying structures cannot be equated (Stearns and Rodrigues, 2020; Zietsch and Sidari, 2020). Nevertheless, the fact remains that individuals of the same species differ from each other regarding LH traits such as maturation rate, reproductive output, and longevity, and that time and energy limitations may be just as true for individuals as are for species (Woodley Menie et al., 2021). According to this, tradeoffs that imply competing allocations between current versus future reproduction might respond to a universal logic and might ubiquitously conform a fast–slow continuum (Figueredo et al., 2005; Del Giudice, 2020). In support of this, evidence of variation along a fast–slow axis within single species has been reported across resource gradients (Singh et al., 2016; Tabak et al., 2018).

Finally, there is no agreement on which traits form part of the fast–slow continuum. This construct was originally circumscribed to a few biodemographic LH traits, such as age at first reproduction, age-specific fertility, and age-specific mortality (Roff, 2002; Del Giudice, 2020), but it has been extended in several ways since then. Particularly important for our work, some additions are aimed to accommodate the construct to humans, who show a number of peculiarities regarding LH: an exceptionally long lifespan, long juvenile and post-reproductive periods, highly altricial offspring, biparental care, and late reproduction, among others (Hill and Kaplan, 1999; Muehlenbein and Flinn, 2011). One such addition is that of mating patterns, which do not usually form part of basic LH traits in evolutionary biology. However, mating is an essential part of reproductive effort, is the stronger predictor of fitness (Kingsolver and Diamond, 2011), and may be particularly important in humans, who are strategic pluralists (Buss and Schmitt, 2019). This means that humans engage in short-term mating, long-term mating, or any combination thereof, with presumably important but still unknown consequences for fitness. For example, it is generally assumed that unrestricted sociosexuality, involving many uncommitted partners, is associated with faster life histories (Belsky, 2012; Xu et al., 2018), but some evidence suggests that may be stability, not number, which boosts reproductive output (Borgerhoff Mulder and Ross, 2019; Međedović, 2020b).

Another approach, the embodied capital theory, focuses instead on somatic effort. It conceives growth/development and maintenance as investments that can be rendered into future reproduction (Hill and Kaplan, 1999; Kaplan et al., 2003). For example, human growth consists largely in acquiring skills, knowledge, and status, which in turn determine wealth, social position, attractiveness and, ultimately, fitness (Von Rueden and Jaeggi, 2016). Also, the ability to gain and maintain access to material resources has proved to have an impact on mating and reproduction across a variety of societies (Hopcroft, 2006; Nettle and Pollet, 2008). The accumulation of social capital—the widest and strongest possible network of family, friends, and allies—may, in an outstandingly social species as ours, crucially determine LH traits such as reproduction and longevity (Kanazawa and Savage, 2009; Lucas and Keller, 2020). As for body maintenance, it enhances reproductive success by preserving health, avoiding damage, and delaying senescence and death. Although these are all future-oriented allocations, their impact on fitness is not so clear in humans. For example, status and resources are more clearly turned into reproductive success in other species than in ours (Meisenberg, 2019), and causal relationships between status, wealth, social capital, condition, mating, and reproductive success have yet to be deciphered (Bolund, 2020).

Finally, other approaches conceive the fast–slow continuum even more broadly. In non-humans, the pace-of-life syndrome (POLS) perspective understands this continuum as a suite of coordinated and co-evolved morphological, physiological, and behavioral traits (Mathot and Frankenhuis, 2018). By way of illustration, slow strategists are expected to hold both greater immune competence and risk aversion than fast-strategists (Lee, 2006; Stamps, 2007), whereas fast strategists should show low immune response and increased levels of boldness, activity, and aggression (Réale et al., 2010). In humans, a sustained effort has been made to link biodemographic LH traits with psychological constructs such as personality and sociosexuality. For example, some fruitful approaches have sought to elucidate how environmental threat, deprivation, or unpredictability during childhood can accelerate the rate of development or can calibrate future reproductive strategies (Belsky et al., 1991; Simpson and Belsky, 2016). It has been established that family disruption, father absence, stepfather presence, and other environmental stressors are associated with earlier age at menarche (Belsky, 2012, 2019; Webster et al., 2014), which in turn is a predictor of advanced sexual debut, sexual risk-taking, earlier pregnancy, and larger numbers of children (Ellis, 2004; Pesonen et al., 2008; Nettle et al., 2011). Also, cues of high local mortality during infancy give rise to discount of the future and can advance reproduction (Wilson and Daly, 1997; Chisholm, 1999; Kruger, 2008). Certainly, these models exhaust neither all the possible determinants of adult reproductive decisions nor the range of personality traits that may accompany fast–slow strategies and have not yet clarified the association of developmental speed with sociosexuality (Ellis, 2004; Pesonen et al., 2008; Richardson et al., 2017; Belsky, 2019). Another influential model, stemming from Differential K theory (Rushton, 1985), proposes that many elements of somatic effort, mating strategy, and personality, covary because they are underlain by a single common factor, denoted as K (Figueredo et al., 2004, 2005). Subjects at the high end of the K-factor report higher levels of family and social support, altruism, religiosity, financial status, planning ability, persistence, and self-directedness. These subjects are hence considered slow strategists, as they appear to prioritize somatic over reproductive effort, parenting over mating effort, and quality over quantity of offspring. The K-factor has been also found to correlate with Covitality, a construct reflecting good general health and well-being, as well as with the General Factor of Personality, a higher-order dimension of socially desirable personality emerging from the covariation between emotional stability, extraversion, conscientiousness, agreeableness, and openness (Figueredo et al., 2007, 2015). As a salient point, the K-factor is claimed to be a measure of slow strategy in and by itself (Black et al., 2017). This has proven controversial, as it consists of psychological variables instead of biodemographic indicators of LH strategy (Copping et al., 2014; Figueredo et al., 2015). Not less important, the existence of a common factor underlying psychological variables does not necessarily entail that this is the case for biodemographic LH traits. For these reasons, the K-factor might be very distant from the biological events it intends to represent (Međedović, 2020a; Sear, 2020). On the whole, support for extended continua such as the POLS and the K-factor is yet far from convincing, whatever the species (Copping et al., 2014; Richardson et al., 2017; Mathot and Frankenhuis, 2018; Royauté et al., 2018; Međedović, 2020a; Sear, 2020).

Even so, personality has attracted particular interest, and its role in LH strategies is increasingly acknowledged in both humans and non-humans (Nettle, 2006; Stamps, 2007; Biro and Stamps, 2008; Réale et al., 2010). It is indeed conceivable that different life strategies—having one mate or many, providing for descendants or leaving them to their fate, saving for the future or living from hand to mouth—also require disparate motivational, emotional, and cognitive machineries, that is, different personalities. In this line, both normal and extreme personalities have been characterized as evolutionary alternative strategies that are successful under certain conditions (Nettle, 2006; Gutiérrez et al., 2013; Del Giudice, 2018). At the same time, caution has been advised not to conflate actual LH traits with their psychological correlates, which can be considered LH-related traits at best (Del Giudice, 2020).

In sum, if some agreement exists on the issue of fast–slow continuum in humans, it is the need of better evidence. The purpose of this study is threefold. First, we want to test how reproductive LH traits are organized in humans. Specifically, we will examine how many axes underlie these traits and whether any of them may be interpreted as a fast–slow continuum. Considering the above, we use biodemographic rather than psychological LH traits, avoid any preconception on the interrelations between traits, analyze each sex separately, and compare unrotated and rotated axes (Del Giudice, 2020; Sear, 2020; Stearns and Rodrigues, 2020). Second, we want to know to what extent these traits are aligned with future-oriented allocations in somatic effort, namely, gaining status, acquiring material resources, accumulating social capital, and maintaining physical and mental health. Finally, we will examine the relationships between LH axes and a broad range of normal and extreme personality traits.

## Materials and Methods

### Subjects

The whole sample consisted of 1,043 outpatients (53.4% female) of mean age 34.7 years (SD 11.0; range 16–74), consecutively referred for personality assessment to the Psychology Service of a general teaching hospital during a 6-year period. The sample was spread along the entire severity range, so that the subjects in the lower quartile of the distribution had normal personalities, whereas those in the upper quartile were severely disordered. Concurrently, 18.3% of them presented a mild to moderate affective disorder, 7.7% an anxiety disorder, 8.9% mixed anxious–depressive symptoms, and 8.0% other psychopathology–substance abuse, eating disorders, somatoform disorders—each with a frequency under 2.5%. Patients presenting a severe affective disorder, psychosis, or dementia were excluded. Diagnoses were made according to the DSM taxonomy (American Psychiatric Association, 2013) by the referring staff and again through clinical interview carried out by two experienced, doctoral level clinical psychologists. A quarter of the sample were currently studying. Among those employed, 19.2% were skilled and 32.3% semiskilled workers. The sample did not differ from the general Spanish population in key parameters such as study level, salary, or average maternal age.^1^ However, it was a younger population (34.7 years vs. 40.2 in Spain) which had not exhausted its reproductive period at the time of assessment (0.5 children vs. 1.3 in Spain). Not all subjects scored all the variables (e.g., income in students), so that actual sample size was between 717 and 1,043 subjects for LH traits except for age at first reproduction (*n* = 317), as two-thirds of the sample were childless. Furthermore, sample size was between 861 and 1,037 for the three personality questionnaires. The study was approved by the ethics committee of the hospital, and all subjects gave informed consent prior to participating.

### Instruments

The *Temperament and Character Inventory* (TCI-R; Cloninger et al., 1994) is a 140-item self-report that operationalizes Cloninger’s Biosocial Model of Personality. It includes four temperament dimensions—novelty seeking, harm avoidance, reward dependence, and persistence—and three character dimensions—self-directedness, cooperativeness, and self-transcendence. Each dimension encompasses three to five narrower traits up to a total of 29. Permissions were obtained to use the TCI-R from the copyright holders. The *Dimensional Assessment of Personality Pathology–Basic Questionnaire* (DAPP-BQ; Livesley and Jackson, 2009) is a self-report consisting of 290 items that measure 18 personality pathology traits. These are structured into four higher order dimensions labeled as emotional dysregulation, inhibition, dissocial behavior, and compulsiveness. The *Personality Diagnostic Questionnaire – 4+* (PDQ–4+; Hyler, 1994) is a 99-item, true–false self-report that assesses the presence and intensity of DSM personality disorders (American Psychiatric Association, 2013). The three questionnaires have shown good psychometric properties in their Spanish versions (Gutiérrez-Zotes et al., 2008, 2015; Calvo et al., 2012). Their 70 dimensions and traits are fully described in Supplementary Table S1, organized into seven empirically based higher-order factors: negative emotionality, persistence, asociality, impulsivity-sensation seeking, antagonism, subordination, and oddity (Gutiérrez et al., 2014).

The *Life Outcome Questionnaire* (LOQ; Gutiérrez et al., 2013) is a self-report questionnaire which assesses a number of life areas such as studies, job, mating, social relationships, finances, and health. Twenty-four variables were selected for this study due to their relevance to LH (Stearns, 1992; Mace, 2000). Specifically, reproductive effort was estimated through the number of produced offspring, age at first reproduction, duration of the longest relationship, lifetime number of mates, and the ratio between short-term mates (<1 year), and total mates. Somatic effort reflects investment in the future through allocations into growth—achieving status, material resources, and social capital—and maintenance/survival. Concretely, status was estimated through more years devoted to studies and later incorporation into the labor market in exchange for higher academic and job levels; acquiring and maintaining material resources included higher income, better balance between revenue and expenditures, less need for external aid (loans), and greater stability in the work place; social capital implied devoting effort to maintain a broad, enduring, and high-quality network of kin and non-kin allies; finally, maintenance was measured through subjective health status, presence of psychopathology, duration of sick leaves, and failures in self-preservation such as suicidal acts or drug abuse, all them predictive of low condition or mortality (Chesney et al., 2014; Ganna and Ingelsson, 2015; Walker et al., 2015). All indicators were assessed on a lifetime basis and appear in full in Table 1. The LOQ has shown adequate criterion validity in previous studies (Gutiérrez et al., 2013; Vall et al., 2015).

**Table 1:** Summary statistics for LH traits and correlations with age and age-corrected residuals.

Life history traits	*n*	Min	Max	Mean	(SD)	r with age	r with residual
**Reproductive effort: mating effort**									
Offspring number (#)	1,041	0	5	0.52	(0.88)	0.54**	0.83**
Age at 1st reproduction (yr)	317	16	57	29.1	(5.8)	0.17**	0.95**
Maximum duration mate (yr)	976	0	44	8.4	(8.4)	0.71**	0.70**
Total mates (#)	873	0	39	4.6	(4.9)	0.06	0.98**
Ratio short/total mates (%)	873	0	100	54.5	(35.6)	−0.35**	0.94**
**Somatic effort: learning/status**									
Education level (1–5)	1,043	0	5	3.3	(1.2)	0.13**	0.96**
Age finished studies (yr)	717	11	57	23.2	(6.9)	0.20**	0.97**
Age began working (yr)	966	6	54	18.5	(3.8)	0.07*	0.98**
Job level (1–3)	739	1	3	1.71	(0.77)	0.21**	0.96**
**Somatic effort: material resources**									
Month income (€)	848	0	6,000	1,268	(1,071)	0.27**	0.95**
Maximum duration job (yr)	942	0	48	8.2	(8.6)	0.72**	0.68**
Wage covers needs (%)	946	0	100	67.7	(30.9)	0.15**	0.98**
Jobs left or fired (#)	723	0	21	2.2	(3.0)	−0.08*	0.98**
Loans (y/n)	925	0	1	0.52	(0.50)	−0.25**	0.96**
**Somatic effort: social capital**									
Family relationships quality (%)	1,038	0	100	63.2	(27.6)	0.03	1.0**
Friends number (#)	1,025	0	21	3.4	(3.0)	−0.03	1.0**
Friend relationships quality (%)	1,033	0	100	64.9	(26.2)	0.00	1.0**
Duration oldest friendship (yr)	1,021	3	63	16.1	(11.6)	0.45**	0.88**
Coworkers relationships quality (%)	962	0	100	63.7	(21.6)	0.08**	1.0**
**Somatic effort: maintenance survival**									
Subjective health (%)	1,028	0	100	64.0	(26.6)	−0.04	1.0**
Drug use (%)	1,025	0	77.3	18.5	(13.6)	−0.14**	0.98**
Suicide attempts (#)	990	0	5	0.53	(1.10)	−0.03	1.0**
Psychopathology (0–10)	951	0	10	4.5	(2.9)	−0.04	1.0**
Sick leaves (months)	868	0	51	6.3	(9.8)	0.32**	0.95**

* *p <* 0.05;

** *p <* 0.01.

### Data Analysis

Reproductive traits are the basic indicators of LH strategy in evolutionary biology (Sear, 2020). Thus, we first tested through principal component analysis (PCA) whether the structure underlying reproductive LH traits—offspring number, age at first reproduction, duration of the longest relationship, total number of mates, and ratio short-term/total mates—took the form of a fast–slow continuum. In a second step, we included the extended set of reproductive and somatic LH traits, i.e., status, material resources, social capital, and maintenance/survival. In both cases, an increasing number of components was successively retained until a singlet emerged. In a final step, the relationships between 70 personality traits and the reproductive LH axes resulting from the first step were examined through Pearson’s correlations and represented graphically.

Two points should be noted concerning PCA. On the one hand, the effect of age was statistically removed from all LH traits through regression, and residuals were used for analysis. This was aimed to control for spuriously inflated correlations between age-dependent variables, such as duration of jobs, income, or offspring number. The associations of the raw LH traits with age and with the resulting age-discounted residuals are shown in Table 1. On the other hand, some variables showed a significant number of missings (e.g., income in students, or age of first reproduction in childless subjects). Given that usual methods—pairwise deletion, listwise deletion, and substitution—are disrecommended because of their important shortcomings (von Hippel, 2004), PCA was based on the matrix of expectation maximization (EM matrix), which provides unbiased maximum likelihood estimates of the correlations (Weaver and Maxwell, 2014).

Although PCA is the commonest method for identifying LH axes, no estimation method is preferable under all circumstances, so we additionally tested Tucker’s Φ congruences with maximum likelihood (ML), weighted least squares (WLS), and principal axis factoring (PAF), which are based on different distributional assumptions (Goretzko et al., 2021). Furthermore, components were left unrotated in a first step, which increases the probability of identifying a general fast–slow continuum explaining the maximum amount of variance (Del Giudice, 2020). Then, as unrotated components may not be biologically interpretable, a second solution was examined after rotating components to varimax. Additionally, given that orthogonal rotations may impose artificial restrictions on phenomena that could be naturally correlated, they were compared as for congruence with oblique rotations (promax and oblimin). Goodness-of-fit of each resulting model was examined through exploratory structural equation modeling (ESEM), which tests the loadings previously obtained through exploratory factor analysis (EFA) within a SEM framework. This approach avoids the unrealistic constraints imposed by usual confirmatory analyses (Marsh et al., 2014). Comparative Fit Index (CFI) and Tucker-Lewis Index (TLI) above 0.95, and Root Mean Square Error of Approximation (RMSEA) and Standardized Root Mean Residual (SRMR) below. 08 were considered good fit. All analyses were repeated in each sex separately, as the optimal solution to a particular LH problem may differ between the sexes. EFA and ESEM were performed in the R packages ‘psych’ (Revelle, 2021) and ‘lavaan’ (Rosseel, 2012), respectively, and the remaining analyses in SPSS v. 25.

## Results

### The Structure of Reproductive LH Traits

#### Principal Component Analysis

Summary statistics for the five reproductive and 19 somatic LH traits are shown in Table 1. The EM matrices of correlations in the whole sample, men, and women are shown, respectively, in Supplementary Tables S2–S4. In a first stage, we undertook PCA of the five reproductive traits alone. Bartlett’s sphericity test was significant (χ^2^ = 584.3, df = 10, *p* < 0.0001), and Kaiser–Meyer–Olkin index was 0.619, indicating that variables are not strongly interrelated but are still adequate for factor analysis. Although one to four components were successively retained and examined for exploratory purposes, usual rules of thumb for the number of factors—Kaiser rule, Sequential Chi-Square Model Tests, Very Simple Structure, and Parallel analysis—agreed in suggesting two components.

#### Unrotated Components

Unrotated and varimax-rotated solutions offered different but complementary solutions. The first unrotated component (Table 2, left) explained 38% of the variance. High scores in this component characterized subjects who start reproduction earlier and show increased reproductive output. It also included more enduring relationships, fewer mates, and a lower short-to-total relationships ratio, so that it was interpreted as a fast continuum with restricted sociosexuality. A second component explaining an additional 24% of variance also reflected advanced reproduction and more offspring, but included more total mates and higher ratio between short-term and total mates. It was considered a fast–slow continuum with unrestricted sociosexuality. Both components were acceptably congruent between the sexes (Tucker’s Φ = 0.96 and 0.92) and across different extraction methods (Φ = 0.91 to 0.99). However, fit was unsatisfactory in ESEM both in the whole sample and in each sex separately. The one- to four-component solutions, together with congruence coefficients and goodness of fit, are shown in Supplementary Table S5.

**Table 2:** Unrotated and varimax-rotated two-component solutions for reproductive LH traits.

	Unrotated		Varimax-rotated	
	Fast-restricted	Fast-unrestricted	Fast-slow	Socio-sexuality
Offspring number	**0.69**	**0.46**	**0.83**	0.01
Age 1st reproduction	**−0.61**	**−0.41**	**−0.74**	−0.01
Max. duration mate	**0.73**	0.01	**0.61**	**−0.39**
Total mates	**−0.35**	**0.75**	0.12	**0.82**
Short/total mates	**−0.62**	**0.51**	−0.23	**0.77**
*Explained variance*	*38%*	*24%*	*34%*	*28%*

Loadings ≥ 0.30 are in boldtype.

#### Rotated Components

**Figure 1:**
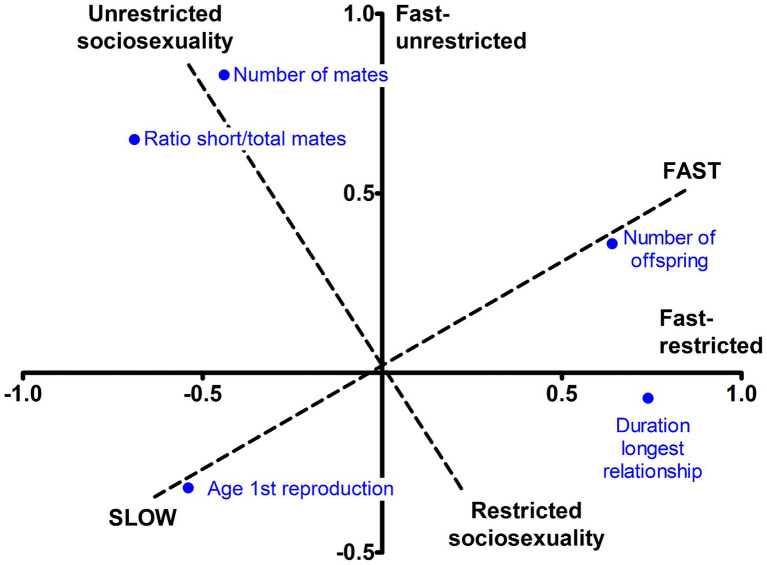
Unrotated and varimax-rotated axes for reproductive LH traits.

We then rotated the two axes to varimax in order to obtain a simpler structure (Table 2, right), which resulted in a 35° turn (Figure 1, dashed lines). The first component, explaining 34% of variance, reflected greater offspring output and earlier reproduction, followed by more enduring relationships, so it can be likened to the fast–slow continuum. The second component reflected more total mates and higher ratio short-to-total mates, with a smaller contribution of shorter relationships duration. It did not include either offspring productivity or age at first birth, so it was considered a restricted–unrestricted sociosexuality continuum. This solution was robust across extraction methods (Φ = 0.96 to 0.99) and across several orthogonal and oblique rotational methods (Φ = 0.99 to 1.00), indicating that the two components were intrinsically unrelated. It also was congruent between the sexes (Φ = 0.98 and 0.95), and obtained excellent fit in ESEM, both in the total sample and in each sex. The one- to four-component rotated solutions, together with congruence coefficients and goodness of fit, are shown in Supplementary Table S6. The unrotated and varimax-rotated two-component solutions obtained separately in men and women are additionally provided in Supplementary Table S7.

### The Joint Structure of Reproductive and Somatic LH Traits

#### Correlations Between Reproductive and Somatic Traits

Somatic LH traits—status attainment, material resources, social capital, and maintenance/survival—maintained rather weak correlations with the four (two unrotated, two rotated) axes previously obtained through PCA (Supplementary Table S8). Fast strategies, and particularly the fast-restricted variant, were significantly but slightly associated with less investment in academic status (mainly in women) but greater resource productivity (only in men), namely, earlier termination of studies (*r* = −0.18), earlier incorporation to the labor market (−0.10), lower academic level (−0.12), higher income (0.13) and more stable jobs (0.13). Unrestricted sociosexuality was associated with job losses (0.16) and drug use (0.15). This pattern of correlations was congruent across sex (Φ = 0.92 to 0.96) and age (Φ = 0.93 to 0.96).

#### Principal Component Analysis

Reproductive and somatic LH traits were then jointly submitted to PCA. Bartlett’s sphericity test was significant (χ^2^ = 5500.0, df = 276, *p* < 0.0001) and Kaiser–Meyer–Olkin index was 0.727, indicating that variables are not strongly interrelated but are still adequate for factor analysis. Usual methods to determine the number of factors did not coincide in this case: Velicer’s MAP suggested three factors, Kaiser rule, Very Simple Structure, and Parallel analysis suggested six, and Sequential Chi-Square Model Test suggested 14. In view of this, one to seven components were successively retained and examined (all solutions in Supplementary Table S9).

#### Unrotated Components

The first unrotated component revealed a continuum underlying most somatic LH traits: more time devoted to attain higher education and job level (status effort), higher earnings, earlier entry to the labor market, greater job stability (resource attainment), broader and better-quality social support network (social capital), and better physical and mental health (maintenance/survival; Table 3, left). This component was highly replicable across method and sex (Φ = 0.97 to 1.00), though it did not achieve acceptable fit in ESEM. Importantly, no indicators of reproductive effort loaded into this component. The second and third components did include reproductive LH traits that adopted the previously found fast-restricted pattern: higher reproductive output, earlier reproduction, and fewer but longer-lasting mates. This configuration was associated with lower status in the second component and with higher status but lower social capital in the third component. The subsequent components were hard to interpret, and none involved the alignment of reproductive and somatic traits into a single continuum (Supplementary Table S9).

**Table 3:** One-component and varimax-rotated seven-component solutions for reproductive and somatic LH traits.

	One component	Varimax-rotated seven components						
		7.5	7.6	7.2	7.3	7.1	7.4	7.7
Offspring number	0.07	**0.81**	0.01	−0.06	0.08	0.04	0.00	−0.10
Age 1st reproduction	0.18	**−0.71**	−0.01	0.14	0.00	0.05	0.26	0.08
Max. duration mate	0.13	**0.60**	**−0.40**	−0.02	0.01	0.04	0.17	0.10
Total mates	−0.05	0.11	**0.76**	−0.01	0.03	0.04	0.03	0.28
Short/total mates	−0.10	−0.24	**0.78**	−0.02	−0.07	0.00	−0.06	−0.13
Education level	**0.51**	−0.06	0.03	**0.87**	0.11	0.08	0.09	−0.01
Job level	**0.46**	0.02	0.01	**0.76**	0.09	0.08	0.10	0.01
Age finished studies	**0.34**	−0.14	−0.01	**0.72**	0.04	0.03	−0.01	−0.06
Age began working	0.22	−0.02	−0.05	**0.55**	−0.21	0.05	0.07	−0.08
Wage covers needs	**0.52**	0.07	0.06	0.12	**0.77**	0.11	0.18	0.17
Month income	**0.54**	0.20	0.14	0.26	**0.66**	0.10	0.24	0.20
Loans	−0.24	0.14	0.02	0.13	**−0.63**	0.03	−0.03	0.13
Max. duration job	0.26	0.09	−0.13	−0.14	**0.53**	0.12	−0.09	−0.23
Jobs left or fired	**−0.47**	−0.01	0.25	−0.19	**−0.46**	−0.25	0.07	**0.30**
Friend rel. Quality	**0.61**	0.05	0.03	0.10	0.06	**0.79**	0.13	−0.05
Friends number	**0.43**	−0.01	0.09	0.08	−0.06	**0.72**	−0.03	−0.04
Coworkers rel. Qual.	**0.57**	0.07	−0.01	0.02	0.19	**0.68**	0.16	0.02
Duration oldest friend	**0.42**	−0.10	−0.06	0.01	0.05	**0.67**	−0.01	0.05
Family rel. Quality	**0.56**	0.07	−0.08	0.06	0.15	**0.48**	0.26	−0.26
Sick leaves	−0.29	−0.02	0.05	−0.11	0.09	0.03	**−0.67**	−0.09
Psychopathology	**−0.59**	0.05	−0.02	−0.02	−0.14	−0.24	**−0.67**	**0.40**
Suicide attempts	**−0.40**	0.16	−0.05	−0.02	−0.06	−0.06	**−0.62**	**0.30**
Subjective health	**0.51**	0.00	−0.04	0.09	0.19	0.21	**0.61**	0.14
Drug use	−0.28	−0.13	0.06	−0.12	−0.05	−0.02	−0.11	**0.79**
*Explained variance*	*16%*	*10%*	*10%*	*9%*	*8%*	*7%*	*6%*	*6%*

Loadings ≥ 0.30 are in boldtype.

#### Rotated Components

Something similar occurred when the axes were rotated to varimax (all solutions in Supplementary Table S10). A fast-restricted continuum was present in the two- and three-component solutions together with lower status or more resource attainment. However, reproductive LH traits formed an independent component from there, and they split into the previously found fast–slow (Φ = 0.97) and sociosexuality continua (Φ = 0.96) in the six- and seven-component solutions (Table 3, right). As the number of retained components increased, somatic effort also branched out into their four constituent elements: gaining status, acquiring resources, accumulating social capital, and maintaining condition. A fast–slow axis bringing reproductive and somatic LH traits together failed to emerge in any case. The six- and seven-component solutions showed acceptable congruence across methods (mean Φ = 0.98, and 0.97, respectively) but not across sex (mean Φ = 0.93, and 0.84). Concerning the latter, the main difference was that relationship duration belonged to the fast–slow continuum in men but to sociosexuality in women. However, fit was good in ESEM, both in the whole sample and in each sex separately (complete results in Supplementary Table S10).

#### Condition-Corrected Principal Component Analysis

Alternatively, the first component in Table 3 could be interpreted as a condition factor, as it encompasses advantages in status, access to material resources, social capital, and physical and mental health. Given that condition can have an effect on tradeoffs (Bolund, 2020; Laskowski et al., 2021), the five reproductive LH traits were submitted to a supplementary PCA after removing the effect of this component through regression analysis. The resulting condition-corrected traits showed almost perfect association with the original traits, from *r* = 0.96 to 1.0. Furthermore, PCA results did not differ from those in Table 2: Congruence was Φ = 1.0 and 0.95 for the unrotated two-component solution and 0.98 and 0.97 for the varimax-rotated solution.

### Reproductive LH Axes and Personality

#### Correlations of Personality Traits With the Fast–Slow and Sociosexuality Axes

**Figure 2:**
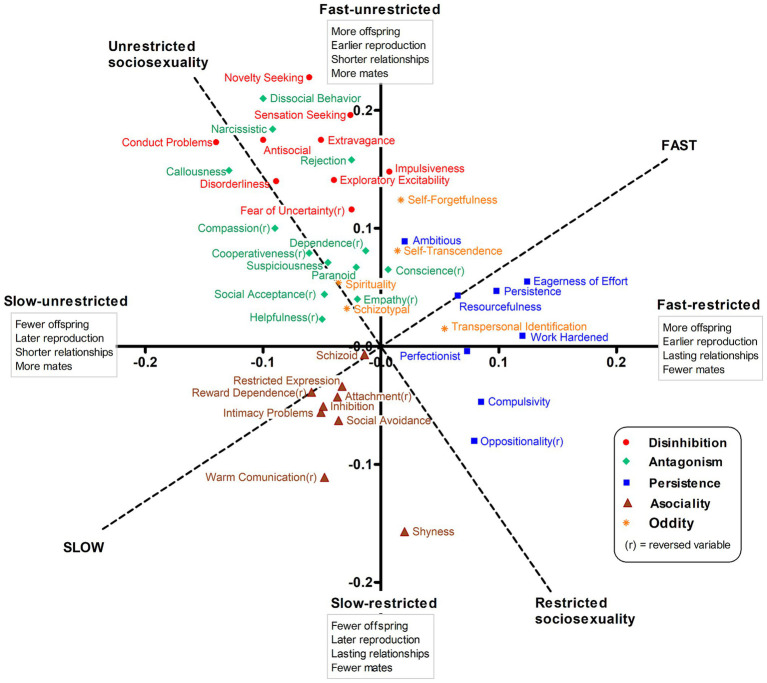
Correlations of personality traits with unrotated and varimax-rotated axes for reproductive LH traits.

Summary statistics for the 70 personality traits are shown in Supplementary Table S11. Figure 2 represents the correlations of personality traits with the unrotated fast-restricted and fast-unrestricted axes (solid lines), and with the 35°-rotated fast–slow and sociosexuality axes (dashed lines) that resulted from the initial PCA only based on reproductive traits. Personality traits reflecting persistence-compulsivity (blue squares) and impulsivity-sensation seeking (red dots) showed the highest association with a fast strategy defined by earlier reproduction and higher reproductive output. Persistence and impulsivity traits differed in sociosexuality, however, so that the former laid along the fast-restricted horizontal axis, while impulsive-sensation seeking traits were aligned with the fast-unrestricted vertical axis. Antagonistic traits (green diamonds) were located along the unrestricted pole of the sociosexuality axis, but were unassociated with the fast–slow continuum. Some oddity traits (orange asterisks), reflecting self-forgetfulness and transpersonal identification, were related to a faster strategy, but less so to sociosexuality. Finally, asocial traits (brown triangles) were distributed across the slow quadrant, reflecting later and lower reproductive output. The two remaining groups of personality traits, reflecting negative emotionality and subordination, were largely unrelated to the LH axes and are not shown (full correlations in Supplementary Table S11 for traits and Supplementary Table S12a for higher-order dimensions; see also Supplementary Figure S1).

#### Congruence of Correlations Across Sex and Age

It is worth noting that associations between LH axes and personality traits were not congruent across sex (Φ = 0.59 to 0.68; Supplementary Table S12b). Concretely, the fast strategy was associated with persistence (*r* = 0.19) and impulsivity (0.13) in men, but with sociability (inverse asociality, −0.10) in women. Sociosexuality was associated with impulsivity in both sexes (0.19 and 0.24), but with antagonism mainly in men (0.15), and with negative emotionality only in women (0.15). On the other hand, whereas the associations between the fast–slow continuum and personality were congruent across age (Φ = 0.96), this was not the case of sociosexuality (Φ = 0.83), which was more closely related to inverse asociality in older than in younger people (r = −0.12 vs. −0.01).

## Discussion

We analyzed in 1,043 outpatients the presence of a fast–slow continuum underlying reproductive LH traits. The first two unrotated components confirmed that subjects can be arranged according to the extent to which they invest in reproductive effort, i.e., advance reproduction and produce more offspring. However, some subjects (fast-restricted) attain this goal through fewer but long-lasting relationships, and others (fast-unrestricted) through a greater number of shorter relationships, shaping a double-track pathway to fast strategy. When rotated, these axes define two orthogonal continua identifiable as fast–slow and sociosexuality. We also found that restricted and unrestricted fast strategies are associated with persistent and disinhibited personality styles, respectively, whereas the slow strategy is linked to asociality.

Our fast–slow axis explains 34% of variance (38% and 24% for unrotated components), which is close to the 37%–45% previously found across mammals (Stearns, 1983; Bielby et al., 2007; Dobson and Oli, 2007; Jeschke and Kokko, 2009; Del Giudice, 2020). However, comparability of our results with mass-adjusted, between-species variance should be taken with caution (Zietsch and Sidari, 2020). On the other hand, whereas reproductive success was chiefly related to advancing reproductive age (*r* = −0.40) in line with previous findings (Sanjak et al., 2018; Međedović, 2020b, 2021), other associations run counter to widespread assumptions. For example, reproductive success is associated with a longer duration of relationships (0.36), but not with having more mates (0.02) or preferring shorter relationships (−0.17). This is unexpected for men according to the Bateman’s third principle, and challenges the available evidence of fast strategies being characterized by a larger number of uncommitted relationships (Figueredo et al., 2005; Xu et al., 2018). However, Bateman’s laws have been called into question before (Tang-Martínez, 2012; Borgerhoff Mulder and Ross, 2019), and having more or shorter relationships is not invariably related to reproductive success (Međedović, 2020b, 2021). Quite the opposite, long-term mating has proven to enhance fitness in humans, especially males, maybe through greater opportunity for copulation (Borgerhoff Mulder and Ross, 2019; Međedović, 2021). The finding of a double-track pathway to fast strategy, based on either more or longer-lasting relationships, may help to integrate these conflicting views, and fits in with a recent theoretical proposal (Del Giudice, 2018).

Concerning the extended analysis of reproductive and somatic LH traits, the first unrotated component shows that most indicators of somatic effort are indeed aligned. This includes investments into: higher status and knowledge, such as higher academic and job level; access to a stable source of material resources through higher income and a regular job career; accumulated social capital in the form of a broader network of family and friends; and a healthier and eventually longer life. These traits are supposed to be rendered into future reproduction and then, typically characterize slow strategists in the evolutionary psychology literature (Figueredo et al., 2007; Del Giudice, 2015). However, two problems become rapidly evident. On the one hand, this component only explains a 16% of the variance, indicating that traits are fairly independent of each other. Thereby, only models that segregate status, resources, social capital, and maintenance/survival attain adequate fit. On the other hand, this axis is not aligned with reproductive traits in any sex, with two exceptions: There are mild associations of fast strategy with higher income in men and with lower academic level in women. Both findings have been reported before (Nettle and Pollet, 2008; Kong et al., 2017), suggesting that greater reproductive success occurs at the cost of status achievement only for women. Thus, in line with some prior proposals (Copping et al., 2014; Richardson et al., 2017; Međedović, 2020a), our results do not support the existence of a unitary axis that brings reproductive and somatic LH traits together, so that examining them separately seems prudent for the time being (Sear, 2020). On the other hand, we do not find a tradeoff between current and future reproduction, a point on which the literature is also inconclusive to date (Högnäs et al., 2017; Bolund, 2020). For example, neither the number of mates, nor status, nor longevity have proven to be clearly related to reproductive output in humans (Bolund, 2020; Međedović, 2020b).

The third relevant finding is that personality traits are weakly but consistently involved in reproductive strategies. The slow strategy, entailing smaller offspring output and later reproduction, is related to asocial personalities, particularly to the absence of warmth and intimacy feelings and to the deployment of an inhibited and avoidant interpersonal style. These subjects are uncomfortable in close relationships and in expressing feelings, which is at odds with the presumption that a slow strategy entails stronger prosocial tendencies and cooperation (Figueredo et al., 2005; Sherman et al., 2013). However, it fits with the proposal of a skilled/provisioning slow subtype characterized by mechanistic cognition, low affiliation, and low agreeableness (Del Giudice, 2018). On the other hand, fast strategies are associated with two different types of personalities, both of which achieve greater reproductive output through advancing reproduction, but are opposite in their sociosexual orientation. Fast-unrestricted subjects tend to act impulsively, prefer novelty and strong stimulation, take risks, break the rules, and are interpersonally opportunistic, selfish, cold, and callous. However, whereas these features are all associated with a greater number of shorter relationships, only impulsivity-sensation seeking, not antagonism, leads to greater reproductive success. This might be, however, at the expense of parenting and survival (Vaurio et al., 2018; Međedović and Petrović, 2019). The second variant of the fast strategy is related to persistence-compulsivity, and particularly to traits implying perseverance despite temptation or frustration, capacity for effort, competence, industriousness, and a high sense of duty. These subjects tend to attain reproductive success through a sociosexually restricted strategy involving fewer and longer relationships, in line with the previous finding that they are preferred for—and better able to—stablishing long-term relationships (Roberts et al., 2007; Buss and Schmitt, 2019; Međedović, 2020b).

Although this second variant had not been reported previously in the literature, it does not collide with standard LH theory, which defines LH strategies through biodemographic indicators according to the seminal formulations by Pianka (1970), Stearns (1992), and Roff (2002). Within evolutionary biology, being fast essentially consists in prioritizing current reproduction—i.e., having more offspring earlier—over any other LH component (Sear, 2020; Stearns and Rodrigues, 2020), and no strong assumptions are made on the accompanying mating strategies or personality traits. In fact, elucidating these relationships has proven to be fraught with difficulties (Mathot and Frankenhuis, 2018; Royauté et al., 2018). The fast-restricted variant does not conflict either with developmental LH approaches (Belsky, 2012; Ellis et al., 2022). These models fundamentally understand fast strategy as an accelerated pace of life, indicated by an advanced onset of menarche, sexual activity, and childbirth. However, a faster pace does not necessarily imply a certain type of personality, nor is axiomatically considered to lead to unrestricted sociosexual orientation (Belsky, 2019). In fact, accelerated development does not consistently covary with either unstable pair bonds, more partners, or lower parental investment (Ellis, 2004), and earlier reproduction or larger numbers of offspring are often associated with longer-lasting marriages (Pesonen et al., 2008; Borgerhoff Mulder and Ross, 2019; Međedović, 2021). Thus, the evidence so far does not limit the range of possible mating strategies and personality traits that can be linked to fast strategy.

Quite the opposite, the finding of an industrious and sexually restricted variant of fast strategy is counterintuitive from the psychometric K-factor model, which is widespread in the evolutionary psychology literature (Figueredo et al., 2004, 2005; Black et al., 2017). Within this framework, normative and restricted subjects are slow strategists, regardless of whether they reproduce earlier or have more offspring, whereas a fast strategist is essentially impulsive, antagonistic, and promiscuous. Discrepancies with the standard LH model are a matter of definition, as psychological variables forming the K-factor are considered the most valid proxies for LH strategies, at least in postindustrial societies (Black et al., 2017). This makes some sense, as recent social and technological transformations—contraceptive use, legally enforced child support, medical advances—may render biodemographic parameters inaccurate indicators of ancestral LH strategies (Figueredo et al., 2014), while the motivational machinery aimed at producing more offspring or prolong life would be unaltered. However, proving that psychological traits outdo biodemographic parameters as indicators of life strategy is a major challenge in itself, so that the debate is ongoing (Copping et al., 2014; Richardson et al., 2017; Međedović, 2020a; Sear, 2020). In any case, both approaches are unanimously seen as largely separated (Black et al., 2017; Nettle and Frankenhuis, 2020), and the associations between their respective measures of LH strategy are in fact “rare, unsystematic, and mostly low in magnitude” (Međedović, 2020a, p. 341).

Finally, we know little about the factors that dictate LH strategic choices. Both LH and personality traits are heritable in part (Stearns et al., 2010; Gavrus-Ion et al., 2017; Paris, 2022), which leaves room for facultative calibration to prevailing ecological conditions (Kuzawa and Bragg, 2012; Chua et al., 2017). For example, different strategies are favored depending on environmental scarcity or abundance, that is, on the intensity of competition for resources (Wilson, 2014). These relationships are not simple, however, and go far beyond the commonly accepted concatenation of environmental harshness, rapid development, future discounting, and unrestricted sexuality (Csathó and Birkás, 2018). Acceleration of the pace of life may result not only from adverse conditions but also from high resource availability, which can be rendered into earlier and higher fecundity (Pettay et al., 2007; Tabak et al., 2018). In this case, abundance could allow greater parental investment, as suggested by better offspring survival (Pettay et al., 2007), or be accompanied by different or even opposite personality features. Furthermore, adversity can sometimes accelerate and other times decelerate developmental pace (Roubinov et al., 2021), or this may depend on the interaction with other ecological conditions. As an illustration, mortality cues lead to risk taking and the desire to reproduce sooner in individuals growing up poor, but to risk avoidance and the desire to delay reproduction in those growing up wealthy (Griskevicius et al., 2011a,b). Still more, the mechanisms that govern strategic choices may be different between the sexes (Hämäläinen et al., 2018) and for each specific LH trait (Flatt and Heyland, 2011). Another often-neglected ecological factor is culture, which was a driving force in the differentiation of human life history from that of higher primates (Richerson and Boyd, 2020). Cultural values and practices can affect virtually every biodemographic LH parameter, from the age of sexual debut to the number and duration of mate relationships to the required investment in each child (Colleran, 2016; Shennan and Sear, 2021). For example, conscientious and dutiful subjects such as our fast-restricted strategists tend to a greater extent to adopt conservative values and be religious (Saroglou, 2010; Gerber et al., 2011; McCann, 2014). This may encourage advanced marriage and childbearing, longer-lasting relationships, and more offspring (Raley, 2020; Međedović, 2021). Lastly, it is worth highlighting that, despite restricted and unrestricted fast strategists are opposite in many respects, the psychological mechanism driving investment in current reproduction might lie in what they share: high levels of energy, proactiveness, ambition, and impatience, as well as an increased drive by incentives and reinforcement (Gutiérrez et al., 2014).

Some caveats should be noted in interpreting our results. First, some controversy exists on the number of axes necessary to explain LH variation. There is no single solution to this problem, as the structure underlying LH traits can be subdivided again and again until the level of individual components forming a hierarchical structure. Even if we have carefully chosen the most interpretable, replicable, and well-fitted level, PCA entails some subjectivity that should be acknowledged. Second, whereas we have taken into account the possible divergences caused by rotational methods (Del Giudice, 2020), it is worth remembering that other methodological decisions may cause changes in the final structure. One of them is the lack in our study of important reproductive LH traits as interbirth interval, parenting effort, or offspring quality. Also, indicators of somatic effort vary considerably in the literature, which may lead to disparate solutions. Third, there are cautions concerning the sample. Most of our subjects have not completed their reproductive period. Furthermore, personality disordered subjects have poorer health, lower socioeconomic status, and lower life expectancy at birth (Fok et al., 2012; Quirk et al., 2016). Even if our sample spanned the full range from healthy to severely disturbed subjects, and if we carried out analyses controlling for age, sex, and condition, our results still need replication in a healthy population.

In conclusion, although there is probably not a unique way to organize LH traits, our results support the existence of a fast–slow and a sociosexuality continua that are replicable across sex and condition. However, they also suggest fleeing simplistic representations: Reproduction, mating, growth, or maintenance do not form a single fast–slow continuum, nor unequivocal tradeoffs between reproductive and somatic effort are apparent. Instead, each component is notably independent of each other. Moreover, the picture is more complex than generally assumed. Fast strategists present with two distinct flavors, meaning that, just as there are multiple pathways to fitness, such as fast and slow strategies, there also are different pathways to a fast strategy, such as restricted and unrestricted sociosexuality. Finally, each strategy is faintly but consistently related to specific personality traits. This does not mean that being risky, or industrious, or asocial are LH strategies in and by themselves. It does suggest, however, that personality traits may reflect the necessary psychological machinery behind strategic LH choices.

## Data Availability Statement

The datasets presented in this study can be found in online repositories. The names of the repository/repositories and accession number(s) can be found at: https://osf.io/xpcyu/?view_only=bae4fc95ec1e47618b87520c466f0adf [Open Science Framework].

## Ethics Statement

This study involves human participants and was reviewed and approved by Comité de Ética de Investigación con Medicamentos (CEIm, Drug Research Ethics Committee), Hospital Clínic, Barcelona, Spain. Written informed consent to participate in this study was provided by the participants’ legal guardian/next of kin.

## Author Contributions

FG, JP, BS, MG, and GV contributed to the conception and design of the study. FG performed the statistical analysis and wrote the first draft of the manuscript. JP, EB, BS, MG, GV, and JR made amendments to the manuscript and rewrote parts of it. All authors recruited samples in their respective centers, assessed outpatients, organized the database, contributed to the manuscript revision, and approved the submitted version.

## Conflict of Interest

The authors declare that the research was conducted in the absence of any commercial or financial relationships that could be construed as a potential conflict of interest.

## Publisher’s Note

All claims expressed in this article are solely those of the authors and do not necessarily represent those of their affiliated organizations, or those of the publisher, the editors and the reviewers. Any product that may be evaluated in this article, or claim that may be made by its manufacturer, is not guaranteed or endorsed by the publisher.

## Footnotes

^1^ www.ine.es
