# Supplementary material for: A Double-Track Pathway to Fast Strategy in Humans and Its Personality Correlates
Source: Front Psychol. 2022 Jun 9;13:889730. doi: 10.3389/fpsyg.2022.889730 (PMC9218359; doi:10.3389/fpsyg.2022.889730)

SUPPLEMENTARY MATERIAL: A DOUBLE-TRACK PATHWAY TO FAST STRATEGY IN HUMANS AND ITS PERSONALITY CORRELATES

Supplementary Table S1. Descriptors for the 70 Personality Scales.

	**Negative Emotionality**	
	DAPP-Emotional Dysregulation	Emotional instability and dysregulated emotional expression; generalized hypersensitivity; pessimism, anhedonia; insecure attachment, submissiveness, fears of rejection and abandonment; unstable and fragmented sense of identity.
(-)	*TCI-Self-Directedness*	Self-determination, purposefulness, internal locus of control; ability to adapt behavior to the demands of a situation in order to achieve personally chosen goals and values.
	TCI-Harm Avoidance	Excessive worrying and pessimism; shyness; tendency to be fearful, doubtful, and easily fatigued.
	DAPP-Identity Problems	Unstable sense of self; feelings of emptiness and boredom; frequent hopelessness and anhedonia.
	DAPP-Anxiousness	Readily feels fearful, worried and threatened; broods about unpleasant experiences; pervasive indecision and guilt.
	DAPP-Affective Lability	Intense and unstable emotions; frequent mood changes; extreme emotional reactions.
	PDQ-Borderline	Very intense and changing emotions; desperately dependent and afraid of abandonment, maintains stormy relationships; impulsive and self-harming behavior; emptiness, instable sense of self.
	TCI-Anticipatory Worry	Tension, pessimistic worrying; anticipates harm and failure, ruminates; difficulties relaxing and getting over humiliating and embarrassing experiences.
(-)	TCI-Purposefulness	Goal-oriented, has a clear sense of meaning and direction, their activities are guided by personal goals and values.
	DAPP-Self-Harm	Engages in deliberate self-harm and has chronic thoughts of suicide.
	DAPP-Cognitive Distortion	Thinking tends to become disorganized especially at times of stress; experiences unusual perceptions and ideas.
	TCI-Fatigability	Asthenic, has less energy than most people, gets very easily tired and needs naps or extra rest periods.
(-)	TCI-Responsibility	Feels free to choose, accepts responsibility for their attitudes and behavior; rarely or no feelings of being a victim of circumstances or external pressure.
	DAPP-Insecure Attachment	Fearful pattern of attachment relationships; afraid of being rejected or abandoned; needs support of significant others to do anything, or seeks proximity when stressed.
	TCI-Sentimentality	Sympathetic, compassionate, easily and deeply moved by sentimental appeals; feels pity and cries easily.
(-)	TCI-Enlightened Second Nature	Has willpower; their habits generally are congruent with, and help to achieve, their long-term values and goals.
	PDQ-Depressive	Mood dominated by dejection and unhappiness; feelings of inadequacy and worthless, low self-esteem, blaming toward self; prone to guilt, pessimism, and worry; also critical and judgmental toward others.

	**Persistence-Compulsivity**	
	TCI-Persistence	Perseverance in spite of fatigue, frustration, and temptations.
	TCI-Work Hardened	Industrious, hard-working; likes challenges and tough jobs; persists despite failure, frustration and fatigue, and do not give up easily.
	TCI-Perfectionist	Thorough, earnest, perfectionist, workaholic; pushes far beyond what is necessary to attain the best possible result.
	TCI-Eagerness of effort	Impatient, gets to work quickly, does not delays duties or hard tasks.
	DAPP-Compulsiveness	Tidy and organized; conscientious; reliable and trustworthy.
	TCI-Ambitious	Aspiring, high-reaching, tenacious; likes to stand out; willing to make major sacrifices to be a success.
	DAPP-Compulsivity	Orderly and systematic; likes structure and organization; concerned about punctuality, schedules, rules, and details; strong sense of duty.
	TCI-Resourcefulness	Efficient, capable; rarely lacks ideas or confidence on how to solve problems; does not wait for others to solve their problems.
(-)	DAPP-Oppositionality	Works slowly, delays unappealing duties; lacks the energy to accomplish tasks that require effort; just drifts through life without setting personal goals.
	PDQ-Obsessive	Needs to keep everything in order and under control; preoccupied with details and rules; unrealistic perfectionism that interferes with tasks; excessively conscientious and ethical; inflexible, rigid; devotion to work and duties with neglect of leisure and friends.

	**Asociality**	
	DAPP-Restricted Expression	Emotionally unresponsive and distant; does not show feelings; uneasy about giving personal information.
	DAPP-Inhibitedness	Introversion, isolation; avoidance of close interpersonal relationships; reluctant to engage in social conversation or express emotions; socially unresponsive and inhibited.
(-)	TCI-Attachment	Close, transparent, prefers intimacy over privacy, seeks the company of friends when worried.
(-)	*TCI-Reward Dependence*	Responds strongly to signals of social reward; seeks social support and approval; warm, sentimental, pleasant, sociable, sensitive, sympathetic.
(-)	TCI-Warm Communication	Affectionate, tender, shares feelings with others.
	TCI-Shyness	Insecure and tense in most social situations, particularly when meeting strangers.
	DAPP-Social Avoidance	Timid, feels insecure with other people and avoids contact unless absolutely necessary; worried about rejection; has poor conversational and social skills.
	DAPP-Intimacy Problems	Lacks interest in, and avoids, intimacy; shows little reaction to separations or reunions; derives little pleasure from sexual relationships.
	PDQ-Schizoid	Finds difficulty forming close relationships; prefers to be alone; does not experience pleasure from people and from most activities; emotionally cold, has little interest in sex or intimacy.

	**Impulsive Sensation-Seeking**	
	TCI- Novelty Seeking	Tendency to be attracted and explore novel stimuli; impulsive decision making; extravagance in approaching reward cues; propensity to lose temper quickly and avoid frustration.
	DAPP-Stimulus Seeking	Needs excitement and stimulation; sensation-seeker, reckless, and impulsive; does not like making plans.
	PDQ-Antisocial	Dangerous or illegal behavior; acts on impulse and gets into risky situations; aggressive, selfish, unempathetic; gets what they wants and does not mind hurting others.
	TCI-Impulsiveness	Takes quick decisions, often on incomplete information; acts on their momentary feelings; controls their impulses poorly.
(-)	TCI-Fear of Uncertainty	Feels tense and anxious in uncertain or unfamiliar circumstances, even when there is little to worry about; cannot tolerate potentially dangerous situations; rarely takes any risks.
	TCI-Disorderliness	Does not like fixed routines and rules; breaks laws they find inconvenient; is a skillful liar.
	TCI-Exploratory Excitability	Enjoys exploring unfamiliar situations or ideas; seeks thrills and excitement; easily bored by monotony and routine.
	DAPP-Conduct Problems	Shows a range of antisocial behaviors and disregard for social norms and laws; resorts to threats or violence; engages in substance misuse.
	TCI-Extravagance	Unrestrained, extravagant with money, prefers spending rather than saving; lives at the limits of their financial capacities.

	**Antagonism**	
	DAPP-Dissocial Behavior	Egocentric and callous, lacks empathy; disregard for social norms and for others; exploitative, sadistic, intimidating, physically violent; juvenile antisocial behavior and substance abuse; unlawful activities; impulsive, sensation-seeker, and reckless.
(-)	*TCI-Cooperativeness*	Agreeable in their relations with other people; identifies with and accept others; fair, tolerant, empathic, helpful, and compassionate.
	DAPP-Callousness	Disregard for the feelings and wellbeing of others; egocentric, lacks empathy and remorse; exploits others and treats them as worthless.
(-)	TCI-Compassion	Forgiving, charitable and benevolent; does not enjoy revenge even if treated badly.
(-)	TCI-Helpfulness	Considerate, supportive; enjoys being in service to others; shares their skills and knowledge so that everyone comes out ahead.
(-)	TCI-Social Acceptance	Tolerant and friendly; accepts other people as they are, even those with very different opinions, values, or appearance.
	DAPP-Rejection	Antagonistic, hostile, and judgmental; thinks to be right, does not admit own errors; dominant, bossy.
(-)	TCI-Pure-Hearted Conscience	Honest, ethical, genuinely conscientious; treats others in a consistently fair manner; uncomfortable if behaves badly towards anyone.
(-)	TCI-Empathy	Tries to imagine themself "in other people's shoes"; attuned to and considerate of other people's feelings; respectful rather than judgmental.
	DAPP-Suspiciousness	Mistrusts others; hyperalert to signs of interpersonal threat and ill-intent; thinks people stand against them.

	PDQ-Paranoid	Suspicious; find it hard to trust others, even friends and family; believes people will take advantage of them; reads threats into neutral situations.
(-)	TCI-Dependence	Seeks to please people; needs emotional support and approval from others; cares deeply how other people regard them.
	PDQ-Narcissistic	Sense of importance and entitlement; requires to be admired and treated as superior; selfish, envious, arrogant; feels upset if ignored; takes advantage of others to get what they wants.
	PDQ-Negativistic	Passive resistance to social and occupational obligations; feels misunderstood or underappreciated; argumentative, often complains about and criticizes authority; resentful against those perceived as more fortunate.
	**Subordination**	
	DAPP-Narcissism	Needs to be liked and recognized, to be the center of attention, or to be approved and accepted; has grandiose fantasies of being admired.
(-)	TCI-Self-Acceptance	Recognizes and accepts both their strengths and limitations; feels comfortable with their actual capacities.
	DAPP-Submissiveness	Subservient, diffident, unassertive; tries to please others; looks to others for guidance and reassurance in taking decisions.
	PDQ-Dependent	Feels needy, weak, unable to make decisions or take responsibilities; delegates important decisions in others; seeks close relationships that provide care, support, and reassurance; feels helpless when alone.
	PDQ-Avoidant	Avoids interpersonal contact because of fears of being criticized or rejected; hypersensitive to disapproval or shame; feels inadequate, unappealing, or inferior.
	PDQ-Histrionic	Dramatic, instable, and overemotional; craves reassurance and attention; is inappropriately seductive in order to be noticed; makes rash decisions; easily influenced by others.
	**Oddity**	
	TCI-Self-Transcendence	Identification of the self with the universe conceived as a unitive whole; expansion of personal boundaries; experiencing spiritual ideas.
	TCI-Self-Forgetfulness	Tends to get absorbed and transcend their self-boundaries when concentrating on what they are doing; appears "absent minded", loses track of time; often feels a sense of wonder or understanding.
	TCI-Spiritual Acceptance	Shows magical thinking, believes in miracles and extrasensory experiences; holds religious or spiritual beliefs; sometimes has felt themself in contact with a divine or supernatural force.
	TCI-Transpersonal Identification	Feels a sense of unity with everything; strong spiritual connection with nature or with people; feels that everything is part of one living organism.
	PDQ-Schizotypal	Experiences distorted thoughts or perceptions; has odd ways of thinking and expressing themself; holds supernatural beliefs; finds making close relationships extremely difficult.

TCI = Temperament and Character Inventory – Revised; DAPP = Dimensional Assessment of Personality Pathology – Basic Questionnaire; PDQ = Personality Diagnostic Questionnaire – 4+.
Personality traits are arranged into seven broad empirically-based factors (Gutiérrez et al., 2014). High-order dimensions of each questionnaire are in *italic*.

Supplementary Table S2. Matrix of Expectation Maximization (EM) Correlations on Which PCA is Based (Whole Sample).

	Offspring number	Age 1st reproduction	Max. duration mate	Total mates	Short/total mates	Education level	Age finished studies	Age began working	Job level	Month income	Max. duration job	Wage covers needs	Jobs left or fired	Loans	Family rel. quality	Friends number	Friend rel. quality	Durat. oldest friend	Coworkers rel. qual.	Subjective health	Drug use	Suicide attempts	Psychopathology	Sick leaves
Offspring number	--																							
Age 1st reproduction	-.40	--																						
Max. duration mate	.36	-.24	--																					
Total mates	.02	.05	-.13	--																				
Short/total mates	-.17	.13	-.33	.32	--																			
Education level	-.08	.17	-.03	-.01	.02	--																		
Age finished studies	-.11	.18	-.05	-.03	.01	.58	--																	
Age began working	-.06	.07	-.05	-.08	.02	.34	.22	--																
Job level	-.05	.12	.03	.05	-.06	.65	.35	.23	--															
Month income	.17	.02	.11	.11	-.03	.27	.13	.06	.23	--														
Max. duration job	.16	-.05	.11	-.05	-.10	-.02	-.01	-.17	-.04	.21	--													
Wage covers needs	.03	-.05	.04	.04	-.06	.16	.07	-.01	.14	.62	.22	--												
Jobs left or fired	-.13	-.04	-.08	.15	.11	-.19	-.09	-.10	-.17	-.20	-.29	-.25	--											
Loans	.02	.01	.00	.03	.01	.01	.02	.10	.00	-.20	-.16	-.33	.19	--										
Family rel. quality	.05	.02	.09	-.08	-.05	.12	.07	.07	.13	.19	.15	.19	-.22	-.06	--									
Friends number	-.01	.00	-.01	.04	.05	.13	.08	.05	.12	.07	.07	.08	-.12	.00	.21	--								
Friend rel. quality	.05	.03	.05	.03	-.02	.15	.13	.08	.12	.17	.07	.20	-.22	-.04	.36	.45	--							
Durat. oldest friend	.01	.13	.06	.00	.01	.09	.01	.06	.09	.16	.13	.09	-.13	-.05	.21	.39	.37	--						
Coworkers rel. qual.	.09	.05	.07	.04	-.05	.11	.04	.02	.11	.19	.11	.24	-.23	-.10	.36	.30	.53	.26	--					
Subjective health	.03	.13	.04	.01	-.05	.17	.09	.06	.12	.22	.03	.26	-.15	-.11	.25	.09	.26	.09	.26	--				
Drug use	-.10	.05	-.06	.20	.06	-.09	-.09	-.08	-.10	-.01	-.10	-.03	.18	.11	-.18	-.05	-.11	-.02	-.07	-.05	--			
Suicide attempts	.04	-.24	-.03	.04	-.03	-.10	-.06	-.05	-.14	-.14	-.08	-.11	.05	.06	-.20	-.07	-.13	-.12	-.08	-.19	.14	--		
Psychopathology	-.01	-.15	-.04	.06	.03	-.14	-.06	-.05	-.12	-.20	-.10	-.19	.19	.12	-.33	-.17	-.27	-.14	-.28	-.35	.29	.47	--	
Sick leaves	.04	-.11	-.08	.01	.03	-.15	-.07	-.12	-.10	-.10	.01	-.06	.00	.02	-.08	-.08	-.06	-.01	-.05	-.28	.06	.21	.28	--

Supplementary Table S3. Matrix of Expectation Maximization (EM) Correlations on Which PCA is Based (Men).

	Offspring number	Age 1st reproduction	Max. duration mate	Total mates	Short/total mates	Education level	Age finished studies	Age began working	Job level	Month income	Max. duration job	Wage covers needs	Jobs left or fired	Loans	Family rel. quality	Friends number	Friend rel. quality	Durat. oldest friend	Coworkers rel. qual.	Subjective health	Drug use	Suicide attempts	Psychopathology	Sick leaves
Offspring number	--																							
Age 1st reproduction	-.47	--																						
Max. duration mate	.34	-.30	--																					
Total mates	.09	-.02	-.05	--																				
Short/total mates	-.18	.25	-.27	.33	--																			
Education level	-.04	.17	-.01	-.01	.01	--																		
Age finished studies	-.04	.15	-.04	-.03	.01	.62	--																	
Age began working	-.10	.17	-.13	-.07	.04	.41	.26	--																
Job level	-.02	.15	-.01	.05	-.06	.64	.37	.25	--															
Month income	.29	-.07	.16	.12	-.05	.28	.09	.09	.22	--														
Max. duration job	.24	-.19	.22	-.08	-.13	-.07	-.02	-.24	-.05	.21	--													
Wage covers needs	.10	-.11	.10	.03	-.09	.13	.00	-.04	.12	.57	.22	--												
Jobs left or fired	-.20	.05	-.07	.08	.14	-.14	-.08	-.05	-.14	-.24	-.38	-.29	--											
Loans	.00	.11	-.01	.00	.03	.03	.06	.14	-.02	-.17	-.08	-.32	.18	--										
Family rel. quality	.04	.03	.04	.00	-.07	.12	.07	.03	.06	.21	.18	.19	-.28	-.03	--									
Friends number	.00	-.03	.05	.06	.04	.10	.11	-.01	.11	.07	.08	.07	-.16	.03	.24	--								
Friend rel. quality	.09	.01	.08	.12	.00	.15	.09	.05	.09	.17	.08	.18	-.19	-.01	.33	.45	--							
Durat. oldest friend	.01	.14	.07	.03	.04	.01	.00	.03	.03	.15	.06	.06	-.03	.02	.22	.41	.35	--						
Coworkers rel. qual.	.11	-.06	.02	.10	-.07	.14	.04	-.02	.09	.23	.11	.30	-.30	-.11	.46	.33	.56	.30	--					
Subjective health	.06	.09	.05	.03	-.07	.18	.10	.07	.04	.16	-.04	.24	-.17	-.08	.25	.10	.37	.12	.35	--				
Drug use	-.10	.07	-.08	.15	.07	-.07	-.07	-.08	-.08	-.06	-.21	.01	.12	.11	-.11	.03	-.06	.03	-.03	-.08	--			
Suicide attempts	-.03	-.24	-.03	.03	-.03	-.10	-.06	-.01	-.10	-.13	-.11	-.12	.11	.04	-.14	-.08	-.12	-.17	-.06	-.22	.16	--		
Psychopathology	-.01	-.15	-.03	.04	.03	-.21	-.10	-.07	-.14	-.19	-.11	-.20	.32	.15	-.31	-.18	-.32	-.20	-.29	-.36	.31	.44	--	
Sick leaves	-.03	-.20	-.11	.07	.06	-.19	-.08	-.11	-.14	-.14	.06	-.09	.02	.08	-.02	-.07	-.08	-.03	-.02	-.22	.09	.20	.27	--

Supplementary Table S4. Matrix of Expectation Maximization (EM) Correlations on Which PCA is Based (Women).

	Offspring number	Age 1st reproduction	Max. duration mate	Total mates	Short/total mates	Education level	Age finished studies	Age began working	Job level	Month income	Max. duration job	Wage covers needs	Jobs left or fired	Loans	Family rel. quality	Friends number	Friend rel. quality	Durat. oldest friend	Coworkers rel. qual.	Subjective health	Drug use	Suicide attempts	Psychopathology	Sick leaves
Offspring number	--																							
Age 1st reproduction	-.34	--																						
Max. duration mate	.37	-.19	--																					
Total mates	-.06	.11	-.21	--																				
Short/total mates	-.14	.10	-.35	.31	--																			
Education level	-.12	.16	-.07	.02	.06	--																		
Age finished studies	-.17	.18	-.07	-.07	.06	.54	--																	
Age began working	-.03	.01	.01	-.08	-.01	.29	.19	--																
Job level	-.09	.13	.04	.06	-.03	.66	.35	.22	--															
Month income	.04	.06	.07	.05	-.02	.30	.20	.04	.31	--														
Max. duration job	.09	-.01	.03	-.02	-.11	.03	.00	-.10	.00	.19	--													
Wage covers needs	-.02	-.02	.01	.04	-.05	.19	.14	.01	.17	.70	.21	--												
Jobs left or fired	-.07	-.06	-.08	.20	.08	-.24	-.12	-.14	-.22	-.19	-.21	-.25	--											
Loans	.03	-.02	.00	.07	.01	-.01	.00	.07	.00	-.24	-.22	-.34	.20	--										
Family rel. quality	.06	.01	.14	-.17	-.05	.12	.06	.11	.19	.18	.12	.19	-.19	-.08	--									
Friends number	-.03	.04	-.08	.00	.08	.15	.05	.09	.12	.09	.07	.08	-.13	-.03	.18	--								
Friend rel. quality	.02	.05	.01	-.05	-.04	.14	.16	.11	.15	.17	.06	.21	-.25	-.06	.38	.46	--							
Durat. oldest friend	.00	.14	.05	-.04	-.01	.15	.02	.09	.16	.17	.20	.11	-.21	-.11	.21	.38	.39	--						
Coworkers rel. qual.	.07	.09	.10	-.06	-.02	.08	.05	.04	.13	.17	.10	.20	-.18	-.10	.29	.26	.52	.23	--					
Subjective health	.02	.09	.06	-.06	-.05	.18	.11	.06	.21	.27	.06	.27	-.15	-.12	.26	.09	.18	.06	.21	--				
Drug use	-.09	.03	-.02	.22	.02	-.09	-.11	-.08	-.09	-.01	-.03	-.08	.21	.11	-.26	-.12	-.17	-.08	-.10	-.06	--			
Suicide attempts	.08	-.25	-.05	.09	-.01	-.13	-.08	-.08	-.20	-.14	-.05	-.09	.02	.06	-.24	-.08	-.15	-.10	-.10	-.14	.17	--		
Psychopathology	-.02	-.13	-.08	.13	.05	-.10	-.07	-.05	-.15	-.19	-.08	-.17	.10	.09	-.35	-.18	-.24	-.10	-.28	-.33	.35	.49	--	
Sick leaves	.09	-.06	-.08	.02	.02	-.12	-.07	-.12	-.11	-.04	-.01	-.01	.02	-.04	-.12	-.08	-.06	.01	-.07	-.30	.07	.19	.28	--

Supplementary Table S5. EM-Based One to Four Unrotated Components for Reproductive LH Traits in the Whole Sample.

	One Component	Two Components		Three Components			Four Components			
	C11	C21	C22	C31	C32	C33	C41	C42	C43	C44
Offspring number	**.69**	**.69**	**.46**	**.69**	**.46**	.03	**.69**	**.46**	.03	.02
Age 1st reproduction	**-.61**	**-.61**	**-.41**	**-.61**	**-.41**	**.56**	**-.61**	**-.41**	**.56**	.12
Max. duration mate	**.73**	**.73**	.01	**.73**	.01	**.48**	**.73**	.01	**.48**	**.39**
Total mates	**-.35**	**-.35**	**.75**	**-.35**	**.75**	**.43**	**-.35**	**.75**	**.43**	**-.34**
Short/total mates	**-.62**	**-.62**	**.51**	**-.62**	**.51**	-.21	**-.62**	**.51**	-.21	**.56**
Explained variance	*38%*	*38%*	*24%*	*38%*	*24%*	*15%*	*38%*	*24%*	*15%*	*12%*
**Congruence with:**										
PAF	1.0	.99	.98	.99	.96	.98	.96	.94	.88	.89
ML	.99	.97	.91	.99	.97	.96	.94	.85	.75	.59
WLS	.99	.99	.94	.98	.95	.98	.98	.96	.98	.99
Congr. men/women	.96	.96	.92	.96	.92	.69	.96	.92	.69	.86
**Fit (whole sample):**										
CFI	.604		.894			.945				.677
TLI	.434		.734			.863				.192
RMSEA	.134		.092			.066				.161
SRMR	.115		.077			.049				.085
**Fit (men/women)**										
CFI	.553/.662		.686/.897			.965/.911				.501/.666
TLI	.362/.517		.552/.853			.913/.779				.000/.166
RMSEA	.148/.118		.124/.065			.054/.080				.206/.155
SRMR	.140/.099		.143/.060			.049/.053				.099/.088

Loadings ≥ .30 are in boldtype. Although PC estimation gives identical loadings in each extraction, PAF, ML and WLS do not, so congruence indices can be slightly different for each solution. PAF = principal axes factoring, ML = maximum likelihood, WLS = weighted least squares, CFI = comparative fit index, TLI = Tucker-Lewis index, RMSEA = root mean square error of approximation, SRMR = standardized root mean residual.

Supplementary Table S6. EM-Based One to Four Varimax-Rotated Components for Reproductive LH Traits in the Whole Sample.

	One Component	Two Components		Three Components			Four Components			
	C11	C21	C22	C31	C33	C32	C43	C41	C42	C44
Offspring number	**.69**	**.83**	.01	**.46**	**.67**	.17	**.64**	**.49**	.16	.08
Age 1st reproduction	**-.61**	**-.74**	-.01	-.01	**-.92**	.13	**-.93**	-.02	.09	-.04
Max. duration mate	**.73**	**.61**	**-.39**	**.85**	.19	-.01	.11	**.93**	-.10	.16
Total mates	**-.35**	.12	**.82**	-.04	-.03	**.93**	-.01	-.06	**.97**	-.16
Short/total mates	**-.62**	-.23	**.77**	**-.64**	.02	**.53**	-.07	-.16	.16	**-.97**
Explained variance	*38%*	*34%*	*28%*	*27%*	*27%*	*24%*	*26%*	*23%*	*20%*	*20%*
**Congruence with:**										
PAF	1.0	.99	.99	.98	.98	.98	.98	.99	.99	1.0
ML	.99	.98	.97	.97	.93	.96	.85	.84	.95	.96
WLS	.99	.98	.96	.98	.97	.97	.99	.99	.99	1.0
Promax	1.0	.99	.99	.99	.97	.99	.97	.96	.99	.97
Oblimin	1.0	1.0	1.0	1.0	.99	.99	.99	.98	.99	.98
Quartimax	1.0	1.0	1.0	1.0	1.0	1.0	1.0	1.0	1.0	1.0
PC unrotated	1.0	.88	.77	.84	.42	.83	.73	.49	.76	.56
Congr. men/women	.96	.98	.95	.88	.91	.97	.87	.84	.99	.96
**Fit (total):**										
CFI	.604		.997			.969				.962
TLI	.434		.985			.895				.810
RMSEA	.134		.022			.058				.078
SRMR	.115		.010			.037				.026
**Fit (men/women)**										
CFI	.553/.662		1.00/.998			.924/.960				.898/.694
TLI	.362/.517		1.00/.992			.746/.865				.492/.000
RMSEA	.148/.118		.000/.016			.093/.062				.132/.210
SRMR	.140/.099		.013/.013			.072/.034				.046/.092

Loadings ≥ .30 are in boldtype. PAF = principal axes factoring, ML = maximum likelihood, WLS = weighted least squares, CFI = comparative fit index, TLI = Tucker-Lewis index, RMSEA = root mean square error of approximation, SRMR = standardized root mean residual.

Supplementary Table S7. EM-Based Unrotated and Varimax-Rotated Two-Component Solutions for Reproductive LH Traits in Men and Women Separately.

	Men (n=379)				Women (n=433)			
	Unrotated		Varimax-Rotated		Unrotated		Varimax-Rotated	
	C21	C22	C21	C22	C21	C22	C21	C22
Offspring number	**.73**	**.37**	**.81**	.09	**.64**	**.53**	**.83**	-.07
Age 1st reproduction	**-.75**	-.24	**-.78**	.04	**-.54**	**-.51**	**-.74**	.00
Max. duration mate	**.69**	.03	**.65**	-.21	**.74**	-.02	**.52**	**-.53**
Total mates	-.15	**.86**	.16	**.86**	**-.49**	**.58**	.05	**.76**
Short/total mates	**-.59**	**.57**	**-.35**	**.74**	**-.63**	**.49**	-.11	**.79**
Explained variance	*39%*	*25%*	*37%*	*27%*	*38%*	*22%*	*30%*	*30%*

Supplementary Table S8. Correlations Between Reproductive LH Axes and Somatic LH Traits.

	Unrotated				Varimax-Rotated			
	Fast-Restricted		Fast-Unrestricted		Fast-Slow		Sociosexuality	
	Total	(♂/♀)	Total	(♂/♀)	Total	(♂/♀)	Total	(♂/♀)
Education level	**-.08***	(-.06/**-.13**)	-.06	(-.07/-.05)	**-.12****	(**-.11**/**-.15**)	-.01	(-.03/.03)
Age finished studies	**-.10***	(-.06/**-.16**)	**-.13****	(-.06/**-.20**)	**-.18****	(-.10/**-.29**)	-.05	(-.02/-.08)
Age began working	-.04	(**-.14**/.02)	**-.08***	(-.10/-.05)	**-.10****	(**-.20**/-.02)	-.04	(-.02/-.05)
Job level	-.03	(-.06/-.04)	-.06	(-.06/-.04)	-.07	(-.09/-.07)	-.03	(-.02/-.01)
Month income	.07	(**.16**/.02)	**.09***	(**.15**/-.01)	**.13****	(**.25**/.02)	.04	(.05/-.02)
Max. duration job	**.16****	(**.30**/.06)	-.03	(-.03/-.04)	**.13****	(**.25**/.03)	**-.10****	(**-.15**/-.06)
Wage covers needs	.04	(**.13**/-.01)	-.01	(.01/-.05)	.03	(**.12**/-.04)	-.03	(-.05/-.03)
Jobs left or fired	**-.16****	(**-.19**/**-.14**)	**.12****	(.05/**.18**)	-.07	(**-.13**/-.01)	**.16****	(**.12**/**.20**)
Loans	-.02	(-.05/-.02)	.05	(.02/.09)	.02	(-.02/.05)	.05	(.04/.08)
Family rel. quality	**.09****	(.06/**.13**)	-.07	(-.03/**-.11**)	.04	(.03/.05)	**-.09****	(-.05/**-.15**)
Friends number	-.05	(-.02/-.07)	.05	(.05/.06)	-.01	(.02/-.03)	.06	(.05/.08)
Friend rel. quality	.03	(.00/.07)	.03	(.10/-.06)	.05	(.08/.03)	.01	(.08/-.07)
Durat. oldest friend	-.01	(-.06/.03)	-.02	(.02/-.05)	-.02	(-.04/-.01)	-.01	(.04/-.05)
Coworkers rel. qual.	**.08***	(.05/**.12**)	.00	(.06/-.06)	**.08***	(.09/.07)	-.03	(.03/**-.10**)
Subjective health	.03	(.07/.04)	-.03	(.01/-.07)	.01	(.06/-.01)	-.04	(-.02/-.08)
Drug use	**-.15****	(**-.19**/-.08)	**.11****	(.08/**.12**)	-.07	(**-.11**/.01)	**.15****	(**.14**/**.13**)
Suicide attempts	.01	(-.03/.01)	.04	(-.01/**.10**)	.04	(-.04/.08)	.03	(.01/.07)
Psychopathology	-.04	(-.06/-.06)	.06	(.04/**.12**)	.00	(-.03/.02)	.06	(.06/**.12**)
Sick leaves	-.02	(**-.12**/.05)	.05	(.07/.06)	.02	(-.06/.09)	.05	(.11/.02)

Significant correlations are in boldtype. * $p < .05$, ** $p < .01$.

Supplementary Table S9. EM-Based One to Seven Unrotated Components for Reproductive and Somatic LH Traits in the Whole Sample.

	One Component	Two Components		Three Components			Four Components			
	C11	C21	C22	C31	C32	C33	C41	C42	C43	C44
Offspring number	.07	.07	**.56**	.07	**.56**	**.31**	.07	**.56**	**.31**	-.06
Age 1st reproduction	.18	.18	**-.53**	.18	**-.53**	-.28	.18	**-.53**	-.28	.04
Max. duration mate	.13	.13	**.47**	.13	**.47**	**.38**	.13	**.47**	**.38**	-.22
Total mates	-.05	-.05	-.16	-.05	-.16	**-.34**	-.05	-.16	**-.34**	**.45**
Short/total mates	-.10	-.10	**-.36**	-.10	**-.36**	**-.45**	-.10	**-.36**	**-.45**	.28
Education level	**.51**	**.51**	**-.55**	**.51**	**-.55**	**.43**	**.51**	**-.55**	**.43**	.07
Age finished studies	**.34**	**.34**	**-.51**	**.34**	**-.51**	**.35**	**.34**	**-.51**	**.35**	.01
Age began working	.22	.22	**-.41**	.22	**-.41**	.27	.22	**-.41**	.27	-.22
Job level	**.46**	**.46**	**-.43**	**.46**	**-.43**	**.40**	**.46**	**-.43**	**.40**	.04
Month income	**.54**	**.54**	.13	**.54**	.13	.17	**.54**	.13	.17	.55
Max. duration job	.26	.26	**.41**	.26	**.41**	.05	.26	**.41**	.05	.21
Wage covers needs	**.52**	**.52**	.21	**.52**	.21	.08	**.52**	.21	.08	**.60**
Jobs left or fired	**-.47**	**-.47**	-.19	**-.47**	-.19	-.19	**-.47**	-.19	-.19	-.04
Loans	-.24	-.24	-.23	-.24	-.23	.02	-.24	-.23	.02	**-.43**
Family rel. quality	**.56**	**.56**	.16	**.56**	.16	-.14	**.56**	.16	-.14	-.20
Friends number	**.43**	**.43**	.01	**.43**	.01	**-.37**	**.43**	.01	**-.37**	-.23
Friend rel. quality	**.61**	**.61**	.10	**.61**	.10	**-.34**	**.61**	.10	**-.34**	-.22
Durat. oldest. friend	**.42**	**.42**	.07	**.42**	.07	**-.34**	**.42**	.07	**-.34**	-.16
Coworkers rel. qual.	**.57**	**.57**	.19	**.57**	.19	-.29	**.57**	.19	-.29	-.09
Subjective health	**.51**	**.51**	-.00	**.51**	-.00	-.09	**.51**	-.00	-.09	.04
Drug use	-.28	-.28	-.11	-.28	-.11	-.19	-.28	-.11	-.19	**.32**
Suicide attempts	**-.40**	**-.40**	.10	**-.40**	.10	.15	**-.40**	.10	.15	.12
Psychopathology	**-.59**	**-.59**	-.03	**-.59**	-.03	.17	**-.59**	-.03	.17	.18
Sick leaves	-.29	-.29	.14	-.29	.14	-.02	-.29	.14	-.02	.12
Variance explained	*16%*	*16%*	*10%*	*16%*	*10%*	*8%*	*16%*	*10%*	*8%*	*7%*
**Congruence with:**										
PAF	1.00	1.00	.97	1.00	.97	.94	1.00	.97	.91	.89
ML	1.00	.87	.52	.80	.50	.48	.82	.51	.56	.69
WLS	1.00	1.00	.97	1.00	.96	.94	1.00	.96	.93	.14
Congr. men/women	.97	.97	.86	.97	.86	.27	.97	.86	.89	.41
**Fit (whole sample):**										
CFI	.628		.779			.860				.912
TLI	.594		.735			.813				.864
RMSEA	.103		.083			.070				.060
SRMR	.099		.078			.064				.052
**Fit (men/women)**										
CFI	.591/.667		.797/.792			.896/.871				.943/.929
TLI	.554/.636		.757/.751			.861/.828				.911/.891
RMSEA	.113/.098		.083/.081			.063/.067				.050/.054
SRMR	.112/.098		.083/.080			.064/.066				.052/.053

Loadings ≥ .30 are in boldtype. PAF = principal axes factoring, ML = maximum likelihood, WLS = weighted least squares, CFI = comparative fit index, TLI = Tucker-Lewis index, RMSEA = root mean square error of approximation, SRMR = standardized root mean residual.

Supplementary Table S9 (cont.). EM-Based One to Seven Unrotated Components for Reproductive and Somatic LH Traits in the Whole Sample.

	Five Components					Six Components					
	C51	C52	C53	C54	C55	C61	C62	C63	C64	C65	C66
Offspring number	.07	**.56**	**.31**	-.06	.20	.07	**.56**	**.31**	-.06	.20	**.35**
Age 1st reproduction	.18	**-.53**	-.28	.04	-.29	.18	**-.53**	-.28	.04	-.29	-.20
Max. duration mate	.13	**.47**	**.38**	-.22	.04	.13	**.47**	**.38**	-.22	.04	**.32**
Total mates	-.05	-.16	**-.34**	**.45**	.24	-.05	-.16	**-.34**	**.45**	.24	**.43**
Short/total mates	-.10	**-.36**	**-.45**	.28	.06	-.10	**-.36**	**-.45**	.28	.06	.04
Education level	**.51**	**-.55**	**.43**	.07	.20	**.51**	**-.55**	**.43**	.07	.20	.00
Age finished studies	**.34**	**-.51**	**.35**	.01	.16	**.34**	**-.51**	**.35**	.01	.16	-.11
Age began working	.22	**-.41**	.27	-.22	.12	.22	**-.41**	.27	-.22	.12	.05
Job level	**.46**	**-.43**	**.40**	.04	.19	**.46**	**-.43**	**.40**	.04	.19	.06
Month income	**.54**	.13	.17	**.55**	.03	**.54**	.13	.17	**.55**	.03	.17
Max. duration job	.26	**.41**	.05	.21	-.05	.26	**.41**	.05	.21	-.05	**-.33**
Wage covers needs	**.52**	.21	.08	**.60**	-.04	**.52**	.21	.08	**.60**	-.04	-.01
Jobs left or fired	**-.47**	-.19	-.19	-.04	-.04	**-.47**	-.19	-.19	-.04	-.04	**.40**
Loans	-.24	-.23	.02	**-.43**	.23	-.24	-.23	.02	**-.43**	.23	**.32**
Family rel. quality	**.56**	.16	-.14	-.20	-.01	**.56**	.16	-.14	-.20	-.01	-.04
Friends number	**.43**	.01	**-.37**	-.23	**.41**	**.43**	.01	**-.37**	-.23	**.41**	-.03
Friend rel. quality	**.61**	.10	**-.34**	-.22	**.33**	**.61**	.10	**-.34**	-.22	**.33**	.02
Durat. oldest. friend	**.42**	.07	**-.34**	-.16	**.31**	**.42**	.07	**-.34**	-.16	**.31**	-.11
Coworkers rel. qual.	**.57**	.19	-.29	-.09	.24	**.57**	.19	-.29	-.09	.24	.03
Subjective health	**.51**	-.00	-.09	.04	-.26	**.51**	-.00	-.09	.04	-.26	**.32**
Drug use	-.28	-.11	-.19	**.32**	.25	-.28	-.11	-.19	**.32**	.25	.28
Suicide attempts	**-.40**	.10	.15	.12	**.52**	**-.40**	.10	.15	.12	**.52**	-.11
Psychopathology	**-.59**	-.03	.17	.18	**.47**	**-.59**	-.03	.17	.18	**.47**	-.10
Sick leaves	-.29	.14	-.02	.12	**.40**	-.29	.14	-.02	.12	**.40**	**-.44**
Variance explained	*16%*	*10%*	*8%*	*7%*	*7%*	*16%*	*10%*	*8%*	*7%*	*7%*	*5%*
**Congruence with:**											
PAF	1.00	.97	.89	.85	.91	1.00	.98	.93	.94	.97	.94
ML	.82	.50	.63	.72	.79	.79	.49	.52	.68	.87	.64
WLS	1.00	.97	.88	.83	.90	1.00	.98	.94	.93	.96	.84
Congr. men/women	.97	.86	.89	.68	.63	.97	.86	.89	.68	.63	.68
**Fit (whole sample):**											
CFI					.963						.980
TLI					.932						.954
RMSEA					.042						.035
SRMR					.037						.030
**Fit (men/women)**											
CFI					.971/.977						.982/.988
TLI					.946/.958						.959/.974
RMSEA					.039/.034						.034/.026
SRMR					.043/.038						.036/.032

Loadings ≥ .30 are in boldtype. PAF = principal axes factoring, ML = maximum likelihood, WLS = weighted least squares, CFI = comparative fit index, TLI = Tucker-Lewis index, RMSEA = root mean square error of approximation, SRMR = standardized root mean residual.

Supplementary Table S9 (cont.). EM-Based One to Seven Unrotated Components for Reproductive and Somatic LH Traits in the Whole Sample.

	Seven Components						
	C71	C72	C73	C74	C75	C76	C77
Offspring number	.07	**.56**	**.31**	-.06	.20	**.35**	**.31**
Age 1st reproduction	.18	**-.53**	-.28	.04	-.29	-.20	**-.30**
Max. duration mate	.13	**.47**	**.38**	-.22	.04	**.32**	-.15
Total mates	-.05	-.16	**-.34**	**.45**	.24	**.43**	.29
Short/total mates	-.10	**-.36**	**-.45**	.28	.06	.04	**.51**
Education level	**.51**	**-.55**	**.43**	.07	.20	.00	.05
Age finished studies	**.34**	**-.51**	**.35**	.01	.16	-.11	.04
Age began working	.22	**-.41**	.27	-.22	.12	.05	.06
Job level	**.46**	**-.43**	**.40**	.04	.19	.06	.04
Month income	**.54**	.13	.17	**.55**	.03	.17	-.03
Max. duration job	.26	**.41**	.05	.21	-.05	**-.33**	.07
Wage covers needs	**.52**	.21	.08	**.60**	-.04	-.01	-.12
Jobs left or fired	**-.47**	-.19	-.19	-.04	-.04	**.40**	-.03
Loans	-.24	-.23	.02	**-.43**	.23	**.32**	.01
Family rel. quality	**.56**	.16	-.14	-.20	-.01	-.04	.08
Friends number	**.43**	.01	**-.37**	-.23	**.41**	-.03	.01
Friend rel. quality	**.61**	.10	**-.34**	-.22	**.33**	.02	-.02
Durat. oldest. friend	**.42**	.07	**-.34**	-.16	**.31**	-.11	-.18
Coworkers rel. qual.	**.57**	.19	-.29	-.09	.24	.03	-.09
Subjective health	**.51**	-.00	-.09	.04	-.26	**.32**	-.20
Drug use	-.28	-.11	-.19	**.32**	.25	.28	**-.56**
Suicide attempts	**-.40**	.10	.15	.12	**.52**	-.11	-.13
Psychopathology	**-.59**	-.03	.17	.18	**.47**	-.10	-.19
Sick leaves	-.29	.14	-.02	.12	**.40**	**-.44**	.13
Variance explained	*16%*	*10%*	*8%*	*7%*	*7%*	*5%*	*4%*
**Congruence with:**							
PAF	1.00	.98	.95	.96	.97	.90	.84
ML	.81	.63	.61	.54	.88	.62	.42
WLS	1.00	.98	.94	.95	.96	.84	.80
Congr. men/women	.97	.86	.89	.68	.63	.68	.20
**Fit (whole sample)**							
CFI							.987
TLI							.961
RMSEA							.032
SRMR							.025
**Fit (men/women)**							
CFI							.991/.993
TLI							.973/.980
RMSEA							.028/.023
SRMR							.030/.027

Loadings ≥ .30 are in boldtype. PAF = principal axes factoring, ML = maximum likelihood, WLS = weighted least squares, CFI = comparative fit index, TLI = Tucker-Lewis index, RMSEA = root mean square error of approximation, SRMR = standardized root mean residual.

Supplementary Table S10. EM-Based One- to Seven-Component Varimax-Rotated Components for Reproductive and Somatic LH Traits in the Whole Sample.

	One Component	Two Components		Three Components			Four Components			
	C11	C21	C22	C31	C32	C33	C41	C42	C43	C44
Offspring number	.07	.18	**.54**	.03	.18	**.62**	-.03	.18	.23	**.58**
Age 1st reproduction	.18	.07	**-.55**	.18	-.29	**-.52**	.21	-.28	-.11	**-.50**
Max. duration mate	.13	.22	**.44**	.02	.05	**.62**	.03	.04	.10	**.65**
Total mates	-.05	-.08	-.15	.08	.10	**-.35**	-.05	.12	.23	**-.52**
Short/total mates	-.10	-.16	**-.33**	.06	.05	**-.58**	.02	.06	-.01	**-.64**
Education level	**.51**	**.39**	**-.64**	.12	**-.86**	.01	.08	**-.85**	.15	-.04
Age finished studies	**.34**	.24	**-.56**	.02	**-.70**	-.04	.02	**-.70**	.04	-.06
Age began working	.22	.14	**-.44**	-.02	**-.53**	-.06	.07	**-.54**	-.18	.04
Job level	**.46**	**.37**	**-.51**	.12	**-.73**	.06	.09	**-.73**	.15	.02
Month income	**.54**	**.56**	.02	**.41**	-.26	**.32**	.13	-.22	**.76**	-.00
Max. duration job	.26	**.34**	**.36**	.29	.13	**.38**	.13	.15	**.44**	.22
Wage covers needs	**.52**	**.55**	.11	**.45**	-.14	**.32**	.14	-.10	**.80**	-.02
Jobs left or fired	**-.47**	**-.50**	-.10	**-.36**	.19	**-.36**	-.26	.18	**-.35**	-.28
Loans	-.24	-.28	-.18	-.26	-.06	-.20	-.05	-.08	**-.53**	.03
Family rel. quality	**.56**	**.58**	.05	**.57**	-.08	.14	**.59**	-.08	.13	.18
Friends number	**.43**	**.42**	-.07	**.54**	.01	-.15	**.60**	.01	-.04	-.07
Friend rel. quality	**.61**	**.62**	-.02	**.71**	-.03	-.03	**.73**	-.03	.09	.03
Durat. oldest. friend	**.42**	**.42**	-.01	**.54**	.04	-.09	**.56**	.04	.04	-.05
Coworkers rel. qual.	**.57**	**.60**	.08	**.67**	.03	.06	**.64**	.03	.22	.05
Subjective health	**.51**	**.50**	-.10	**.48**	-.19	.05	**.42**	-.18	.25	-.01
Drug use	-.28	**-.30**	-.05	-.18	.16	-.26	-.25	.17	.06	**-.36**
Suicide attempts	**-.40**	**-.38**	.18	**-.40**	.17	.09	**-.42**	.17	-.03	.05
Psychopathology	**-.59**	**-.59**	.08	**-.59**	.16	-.04	**-.61**	.16	-.11	-.07
Sick leaves	-.29	-.26	.19	-.21	.24	.02	-.24	.25	.02	-.02
Explained variance	*16%*	*16%*	*10%*	*14%*	*10%*	*9%*	*12%*	*10%*	*9%*	*8%*
**Congruence with:**										
PAF	1.00	1.00	.96	1.00	.98	.99	1.00	.98	.98	.98
ML	1.00	.96	.75	.95	.97	.59	.99	.96	.97	.92
WLS	1.00	1.00	.95	1.00	.98	.99	.91	.96	.50	.85
Promax	---	1.00	.99	.98	.99	.99	.98	.98	.98	1.00
Oblimin	---	.97	.99	.99	.99	1.00	.99	.99	.98	1.00
Quartimax	---	.99	.97	1.00	1.00	1.00	1.00	1.00	1.00	1.00
Congr. men/women	.97	.98	.88	.79	.67	.70	.85	.70	.93	.18
**Fit (whole sample):**										
CFI	.628		.781			.859				.912
TLI	.594		.736			.810				.864
RMSEA	.103		.083			.071				.060
SRMR	.099		.078			.064				.052
**Fit (men/women)**										
CFI	.591/.667		.797/.799			.895/.871				.943/.929
TLI	.554/.636		.756/.758			.858/.825				.911/.891
RMSEA	.113/.098		.083/.080			.064/.068				.050/.054
SRMR	.112/.098		.083/.079			.064/.066				.052/.053

Loadings ≥ .30 are in boldtype. PAF = principal axes factoring, ML = maximum likelihood, WLS = weighted least squares, CFI = comparative fit index, TLI = Tucker-Lewis index, RMSEA = root mean square error of approximation, SRMR = standardized root mean residual.

Supplementary Table S10 (cont.). EM-Based One- to Seven-Component Varimax-Rotated Components for Reproductive and Somatic LH Traits in the Whole Sample.

	Five Components					Six Components					
	C51	C52	C54	C55	C53	C61	C62	C63	C64	C65	C66
Offspring number	.10	-.11	.20	.22	**.59**	.07	-.08	.09	.01	**.75**	.03
Age 1st reproduction	.00	.18	-.07	**-.41**	**-.52**	.02	.16	-.01	-.26	**-.65**	-.02
Max. duration mate	.05	-.02	.09	.02	**.65**	.02	.00	.00	-.13	**.71**	-.15
Total mates	.10	-.05	.22	.29	**-.51**	.07	-.02	.05	-.02	-.07	**.75**
Short/total mates	.06	-.05	.00	.08	**-.64**	.06	-.05	-.04	.02	**-.47**	**.44**
Education level	.08	**.87**	.13	-.08	-.04	.08	**.87**	.11	-.09	-.06	.00
Age finished studies	.02	**.72**	.02	-.06	-.06	.03	**.71**	.04	.01	-.14	-.07
Age began working	.06	**.55**	-.19	-.10	.03	.06	**.54**	-.21	-.07	-.02	-.09
Job level	.09	**.75**	.13	-.06	.01	.09	**.76**	.09	-.10	.02	.01
Month income	.11	.24	**.76**	-.04	.01	.09	.27	**.66**	-.22	.19	.26
Max. duration job	.11	-.15	**.45**	-.04	.23	.13	-.15	**.54**	.07	.09	-.24
Wage covers needs	.10	.10	**.81**	-.07	-.01	.10	.13	**.77**	-.17	.09	.16
Jobs left or fired	-.23	-.19	**-.34**	.13	-.28	-.26	-.18	**-.46**	-.04	-.04	**.39**
Loans	.05	.13	**-.55**	.14	.03	.03	.13	**-.63**	.04	.14	.12
Family rel. quality	**.48**	.05	.14	**-.33**	.17	**.49**	.05	.15	-.29	.07	-.21
Friends number	**.72**	.08	-.06	.02	-.06	**.72**	.08	-.06	.02	-.04	.04
Friend rel. quality	**.79**	.09	.08	-.12	.03	**.79**	.10	.06	-.14	.05	.00
Durat. oldest. friend	**.65**	.02	.03	-.03	-.04	**.65**	.02	.05	.01	-.06	-.02
Coworkers rel. qual.	**.67**	.02	.21	-.12	.05	**.67**	.02	.18	-.16	.09	.02
Subjective health	.21	.09	.28	**-.45**	-.02	.18	.10	.17	**-.59**	.06	.09
Drug use	-.06	-.09	.04	**.39**	**-.35**	-.08	-.07	-.07	.18	-.02	**.57**
Suicide attempts	-.07	-.01	-.09	**.68**	.08	-.07	.00	-.06	**.65**	.18	.16
Psychopathology	-.26	.00	-.17	**.73**	-.05	-.25	.00	-.15	**.71**	.08	.23
Sick leaves	.03	-.12	-.02	**.51**	-.00	.06	-.13	.10	**.66**	-.07	-.05
Explained variance	*10%*	*10%*	*9%*	*9%*	*8%*	*10%*	*10%*	*9%*	*9%*	*8%*	*7%*
**Congruence with:**											
PAF	.99	.99	.98	.99	.99	.99	.99	.98	.97	.99	.98
ML	.99	.98	.97	.98	.96	.99	.98	.94	.94	.94	.93
WLS	.99	.98	.98	.99	.99	.99	.99	.97	.96	.98	.95
Promax	.97	.99	.97	.98	.99	.97	.99	.97	.97	.99	.99
Oblimin	.98	.99	.99	.99	1.00	.99	.99	.99	.98	1.00	.99
Quartimax	1.00	1.00	1.00	1.00	1.00	1.00	1.00	.99	1.00	1.00	.99
Congr. men/women	.92	.97	.89	.92	.90	.96	.97	.93	.90	.91	.91
**Fit (whole sample):**											
CFI					.963						.980
TLI					.932						.954
RMSEA					.042						.035
SRMR					.037						.030
**Fit (men/women)**											
CFI					.971/.929						.982/.977
TLI					.946/.891						.959/.958
RMSEA					.039/.054						.034/.034
SRMR					.043/.053						.036/.038

Loadings ≥ .30 are in boldtype. PAF = principal axes factoring, ML = maximum likelihood, WLS = weighted least squares, CFI = comparative fit index, TLI = Tucker-Lewis index, RMSEA = root mean square error of approximation, SRMR = standardized root mean residual.

Supplementary Table S10 (cont.). EM-Based One- to Seven-Component Varimax-Rotated Components for Reproductive and Somatic LH Traits in the Whole Sample.

	Seven Components						
	PA1	PA2	PA4	PA5	PA3	PA7	PA6
Offspring number	.04	-.06	.08	.00	**.81**	.01	-.10
Age 1st reproduction	.05	.14	.00	.26	**-.71**	-.01	.08
Max. duration mate	.04	-.02	.01	.17	**.60**	**-.40**	.10
Total mates	.04	-.01	.03	.03	.11	**.76**	.28
Short/total mates	.00	-.02	-.07	-.06	-.24	**.78**	-.13
Education level	.08	**.87**	.11	.09	-.06	.03	-.01
Age finished studies	.03	**.72**	.04	-.01	-.14	-.01	-.06
Age began working	.05	**.55**	-.21	.07	-.02	-.05	-.08
Job level	.08	**.76**	.09	.10	.02	.01	.01
Month income	.10	.26	**.66**	.24	.20	.14	.20
Max. duration job	.12	-.14	**.53**	-.09	.09	-.13	-.23
Wage covers needs	.11	.12	**.77**	.18	.07	.06	.17
Jobs left or fired	-.25	-.19	**-.46**	.07	-.01	.25	**.30**
Loans	.03	.13	**-.63**	-.03	.14	.02	.13
Family rel. quality	**.48**	.06	.15	.26	.07	-.08	-.26
Friends number	**.72**	.08	-.06	-.03	-.01	.09	-.04
Friend rel. quality	**.79**	.10	.06	.13	.05	.03	-.05
Durat. oldest. friend	**.67**	.01	.05	-.01	-.10	-.06	.05
Coworkers rel. qual.	**.68**	.02	.19	.16	.07	-.01	.02
Subjective health	.21	.09	.19	**.61**	.00	-.04	.14
Drug use	-.02	-.12	-.05	-.11	-.13	.06	**.79**
Suicide attempts	-.06	-.02	-.06	**-.62**	.16	-.05	**.30**
Psychopathology	-.24	-.02	-.14	**-.67**	.05	-.02	**.40**
Sick leaves	.03	-.11	.09	**-.67**	-.02	.05	-.09
Explained variance	*10%*	*10%*	*9%*	*8%*	*7%*	*6%*	*6%*
**Congruence with:**							
PAF	.99	.99	.97	.98	.98	.98	.94
ML	.99	.98	.91	.94	.98	.92	.48
WLS	.99	.98	.97	.98	.97	.98	.94
Promax	.97	.99	.97	.97	.98	.99	.98
Oblimin	.99	.99	.99	.98	.99	1.00	.99
Quartimax	1.00	1.00	.99	.99	1.00	1.00	.98
Congr. men/women	.95	.98	.90	.88	.79	.71	.67
**Fit (whole sample)**							
CFI							.987
TLI							.961
RMSEA							.032
SRMR							.025
**Fit (men/women)**							
CFI							.991/.988
TLI							.973/.974
RMSEA							.028/.026
SRMR							.030/.032

Loadings ≥ .30 are in boldtype. PAF = principal axes factoring, ML = maximum likelihood, WLS = weighted least squares, CFI = comparative fit index, TLI = Tucker-Lewis index, RMSEA = root mean square error of approximation, SRMR = standardized root mean residual.

Supplementary Table S11. Summary Statistics of the 70 Personality Traits, and Their Correlations with Unrotated and Varimax-Rotated Basic LH Axes.

Personality Traits	Mean	(SD)	Correlations with Basic LH Axes: Fast-Restricted	Fast-Unrestric-ted	Fast-Slow	Socio-sexuality
**Negative Emotionality**						
DAPP-Emotional Dysregulation	452.4	(106.2)	-.01	.04	.02	.04
TCI-Self-directedness (-)	127.5	(24.3)	-.06	.05	-.03	.07
TCI-Harm Avoidance	110.7	(21.1)	-.03	**-.10****	**-.10***	-.06
DAPP-Identity Problems	47.9	(15.6)	.00	.02	.01	.02
DAPP-Anxiousness	52.4	(15.0)	.00	.02	.01	.02
DAPP-Affective Lability	51.5	(13.8)	.01	**.08***	.06	.06
PDQ-Borderline	4.2	(2.3)	.01	**.09***	.07	.07
TCI-Anticipatory Worry	35.6	(8.1)	-.05	-.04	**-.08***	-.01
TCI-Purposefulness (-)	18.2	(5.7)	-.06	.01	-.05	.04
DAPP-Self-Harm	23.9	(13.4)	-.02	.04	.01	.04
DAPP-Cognitive Distortion	38.1	(13.2)	.02	**.08***	**.08***	.05
TCI-Fatigability	26.7	(7.0)	-.06	-.01	-.07	.02
TCI-Responsibility (-)	27.4	(6.7)	.03	.03	.06	.01
DAPP-Insecure Attachment	46.9	(15.0)	**.08***	.01	**.08***	-.03
TCI-Sentimentality	29.3	(5.0)	.07	-.03	.05	-.05
TCI-Enlightened Second Nature (-)	34.3	(8.1)	**-.08***	.05	-.05	.07
PDQ-Depressive	4.5	(1.9)	-.01	-.03	-.03	-.02
**Persistence-Compulsivity**						
TCI-Persistence	106.3	(22.5)	**.10***	.05	**.13****	-.01
TCI-Work Hardened	25.0	(6.4)	**.12****	.01	**.12****	-.05
TCI-Perfectionist	24.8	(6.6)	.07	.00	.07	-.04
TCI-Eagerness of effort	28.4	(6.5)	**.12****	.06	**.16****	-.01
DAPP-Compulsiveness	102.9	(20.6)	**.10****	-.07	.05	**-.10****
TCI-Ambitious	28.2	(7.6)	.02	**.09***	**.08***	.06
DAPP-Compulsivity	52.0	(12.3)	**.09***	-.05	.05	**-.08***
TCI-Resourcefulness	15.7	(4.6)	.07	.04	**.09***	.00
DAPP-Oppositionality (-)	45.6	(13.0)	**.08***	**-.08***	.02	**-.10****
PDQ-Obsessive	3.9	(1.7)	-.01	.05	.02	.04
**Asociality**						
DAPP-Restricted Expression	42.2	(12.2)	-.03	-.03	-.05	-.01
DAPP-Inhibitedness	74.9	(19.4)	-.05	-.05	**-.08***	-.02
TCI-Attachment (-)	20.6	(5.8)	-.04	-.04	-.07	-.02
TCI-Reward Dependence (-)	104.4	(17.0)	-.06	-.04	**-.08***	.00
TCI-Warm Communication (-)	34.1	(7.8)	-.05	**-.11****	**-.12****	-.06
TCI-Shyness	22.8	(6.5)	.02	**-.16****	**-.09***	**-.13****
DAPP-Social Avoidance	44.4	(13.9)	-.04	-.06	**-.08***	-.03
DAPP-Intimacy Problems	33.3	(10.2)	-.05	-.06	**-.09***	-.02
PDQ-Schizoid	2.1	(1.5)	-.01	-.01	-.02	.00

**Impulsive Sensation-Seeking**						
TCI- Novelty Seeking	104.6	(16.4)	-.06	**.23****	**.10****	**.20****
DAPP-Stimulus Seeking	40.8	(12.1)	-.03	**.20****	**.11****	**.16****
PDQ-Antisocial	1.5	(1.6)	**-.10****	**.18****	.03	**.18****
TCI-Impulsiveness	24.2	(6.2)	.01	**.15****	**.11****	**.11****
TCI-Fear of Uncertainty (-)	25.7	(5.4)	-.03	**.12****	.06	**.10****
TCI-Disorderliness	19.5	(4.7)	**-.09***	**.14****	.01	**.15****
TCI-Exploratory Excitability	31.3	(6.1)	-.04	**.14****	.06	**.13****
DAPP-Conduct Problems	28.1	(10.1)	**-.14****	**.17****	-.01	**.20****
TCI-Extravagance	29.6	(7.3)	-.05	**.18****	.07	**.16****
**Antagonism**						
DAPP-Dissocial Behavior	185.0	(41.6)	**-.10****	**.21****	.05	**.21****
TCI-Cooperativeness (-)	135.8	(18.2)	-.06	**.08***	.00	**.09***
DAPP-Callousness	32.7	(9.5)	**-.13****	**.15****	-.02	**.17****
TCI-Compassion (-)	27.1	(6.4)	**-.09***	**.10****	-.02	**.12****
TCI-Helpfulness (-)	30.5	(4.4)	-.05	.02	-.03	.04
TCI-Social Acceptance (-)	29.7	(5.5)	-.05	.04	-.02	.06
DAPP-Rejection	42.6	(11.2)	-.03	**.16****	**.08***	**.13****
TCI-Pure-Hearted Conscience (-)	30.2	(4.8)	.01	.07	.05	.05
TCI-Empathy (-)	18.3	(3.5)	-.02	.04	.01	.04
DAPP-Suspiciousness	32.1	(11.6)	-.05	.07	.01	**.08***
PDQ-Paranoid	2.7	(1.9)	-.02	.07	.03	.06
TCI-Dependence (-)	20.4	(4.1)	-.01	**.08***	.04	.07
PDQ-Narcissistic	2.4	(1.8)	**-.09****	**.18****	.04	**.18****
PDQ-Negativistic	2.6	(1.6)	-.05	**.09***	.02	**.09***
**Subordination**						
DAPP-Narcissism	44.7	(13.4)	**-.09***	**.08***	-.03	**.10****
TCI-Self-Acceptance (-)	31.8	(8.4)	-.05	**.09***	.01	**.09***
DAPP-Submissiveness	41.5	(12.5)	-.01	-.01	-.02	.00
PDQ-Dependent	2.8	(2.3)	-.01	.03	.01	.03
PDQ-Avoidant	3.6	(2.0)	-.04	-.06	**-.08***	-.03
PDQ-Histrionic	2.6	(1.7)	-.04	**.15****	**.07***	**.13****
**Oddity**						
TCI-Self-Transcendence	63.2	(17.6)	.01	**.08***	.07	.06
TCI-Self-Forgetfulness	27.6	(7.8)	.02	**.12****	**.10****	**.09***
TCI-Spiritual Acceptance	17.5	(6.8)	-.04	.05	.00	.06
TCI-Transpersonal Identification	18.1	(6.5)	.05	.01	.06	-.01
PDQ-Schizotypal	3.2	(2.1)	-.03	.03	.00	.04

* $p < .05$, ** $p < .01$. Significant coefficients are in boldtype.
(-) = The scale is reversed with respect to the factor. Higher-order dimensions of each instrument are in italics.

Supplementary Table S12a. Correlations of the Seven Higher-Order Dimensions of Personality with the Basic LH Axes in the Whole Sample (n = 678).

	FR	FU	FS	SO
Negative Emotionality	.01	.02	.02	.01
Persistence-Compulsivity	**.10****	.04	**.13****	-.01
Asociality	-.04	**-.10****	**-.11****	-.06
Impulsive Sensation-Seeking	**-.08***	**.24****	**.09***	**.22****
Antagonism	**-.09***	**.13****	.00	**.14****
Subordination	-.06	.03	-.04	.05
Oddity	.02	**.10***	**.09***	.07

* $p < .05$, ** $p < .01$. Significant coefficients are in boldtype.
FR=Fast-Restricted; FU=Fast-Unrestricted; FS=Fast-Slow; SO=Sociosexuality.

Supplementary Table S12b. Correlations of the Seven Higher-Order Dimensions of Personality with the Basic LH Axes in Subgroups of Men-Women and Younger-Older.

	Men (n = 318)				Women (n = 360)				Younger (n=381)				Older (n=297)			
	FR	FU	FS	SO	FR	FU	FS	SO	FR	FU	FS	SO	FR	FU	FS	SO
Negative Emotionality	.03	-.09	-.04	-.08	-.06	**.16****	.05	**.15****	.07	-.02	.06	-.04	-.04	.07	.01	.08
Persistence-Compulsivity	.11	**.12***	**.19****	.05	.09	-.03	.06	-.06	.08	.03	**.14****	-.01	**.13***	.06	**.15***	-.02
Asociality	.01	**-.13***	-.09	-.10	-.04	-.10	**-.10***	-.06	-.06	-.04	**-.12***	-.01	-.03	**-.17****	**-.12***	**-.12***
Impulsive Sensation-Seeking	-.03	**.23****	**.13***	**.19****	**-.11***	**.25****	.06	**.24****	**-.14****	**.22****	**.10***	**.21****	-.07	**.29****	.10	**.26****
Antagonism	-.06	**.16****	.06	**.15****	-.07	.07	-.03	.08	**-.12***	**.14****	.02	**.15****	-.08	.11	-.01	**.13***
Subordination	-.09	-.01	-.09	.03	-.05	.08	.00	.09	-.04	.01	-.04	.02	-.10	.05	-.06	.10
Oddity	.03	.10	.10	.06	-.01	**.11***	.07	.09	.00	.11	**.12***	.08	.04	.09	.08	.05

* $p < .05$, ** $p < .01$. Significant coefficients are in boldtype.
FR=Fast-Restricted; FU=Fast-Unrestricted; FS=Fast-Slow; SO=Sociosexuality.

Supplementary Figure S1. Personality Dimensions Unrelated to Basic LH Axes: Negative Emotionality and Subordination

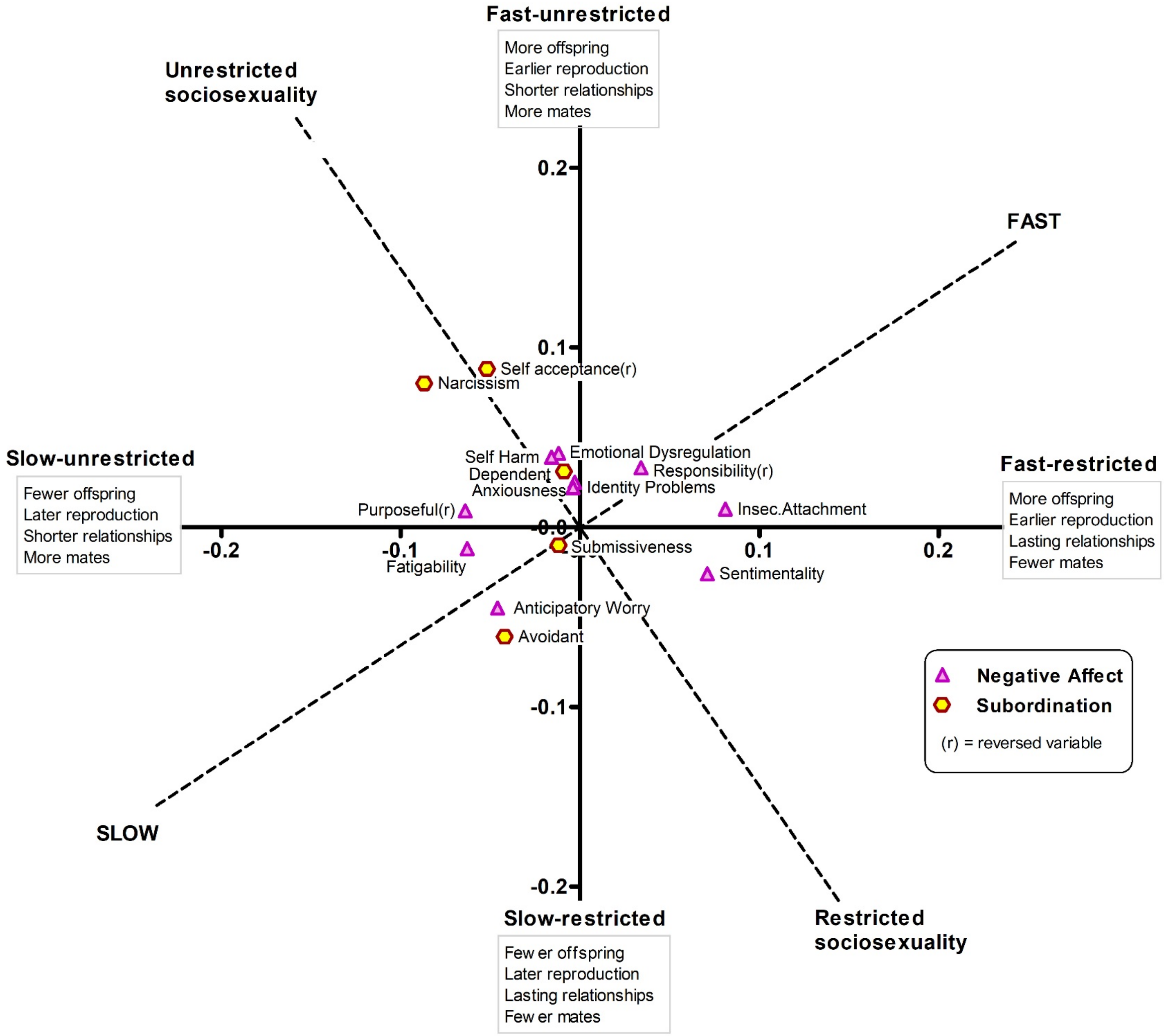

Supplement: Supplementary file 1 [file Data_Sheet_1.PDF]
